# Global Transcriptional Profiling of the Cyanobacterium *Chlorogloeopsis fritschii* PCC 9212 in Far-Red Light: Insights Into the Regulation of Chlorophyll *d* Synthesis

**DOI:** 10.3389/fmicb.2019.00465

**Published:** 2019-03-13

**Authors:** Ming-Yang Ho, Donald A. Bryant

**Affiliations:** ^1^Department of Biochemistry and Molecular Biology, The Pennsylvania State University, University Park, PA, United States; ^2^Intercollege Graduate Degree Program in Plant Biology, The Pennsylvania State University, University Park, PA, United States; ^3^Department of Chemistry and Biochemistry, Montana State University, Bozeman, MT, United States

**Keywords:** far-red light photoacclimation, photosystem I, photosystem II, phycobilisome, photosynthesis, erythromycin, transcription profiling, RNAseq

## Abstract

Some terrestrial cyanobacteria can acclimate to and then utilize far-red light (FRL; λ = 700–800 nm) to perform oxygenic photosynthesis through a process called Far-Red Light Photoacclimation (FaRLiP). During FaRLiP, cells synthesize chlorophylls (Chl) *d* and Chl *f* and extensively remodel their photosynthetic apparatus by modifying core subunits of photosystem (PS)I, PSII, and the phycobilisome (PBS). Three regulatory proteins, RfpA, RfpB, and RfpC, are encoded in the FaRLiP gene cluster; they sense FRL and control the synthesis of Chl *f* and expression of the FaRLiP gene cluster. It was previously uncertain if Chl *d* synthesis and other physiological and metabolic changes to FRL are regulated by RfpABC. In this study we show that Chl *d* synthesis is regulated by RfpABC; however, most other transcriptional changes leading to the FRL physiological state are not regulated by RfpABC. Surprisingly, we show that erythromycin induces Chl *d* synthesis *in vivo*. Transcriptomic and pigment analyses indicate that thiol compounds and/or cysteine proteases could be involved in Chl *d* synthesis in FRL. We conclude that the protein(s) responsible for Chl *d* synthesis is/are probably encoded within the FaRLiP gene cluster. Transcriptional responses to FRL help cells to conserve and produce energy and reducing power to overcome implicit light limitation of photosynthesis during the initial acclimation process to FRL.

## Introduction

Photosynthetically active radiation for oxygenic photosynthesis by cyanobacteria was long-believed to be limited to visible light (λ = 400 to 700 nm). However, the discoveries of Chl *d* in *Acaryochloris marina*, Chl *f* in *Halomicronema hongdechloris*, and FaRLiP in terrestrial cyanobacteria have demonstrated that the wavelength range for oxygenic photosynthesis for some cyanobacteria actually extends well into the far-red/near infrared wavelength region of the solar spectrum (FRL; λ = 700 to ∼800 nm) (Miyashita et al., 1996; Chen et al., 2010; Chen, 2014; Gan et al., 2014; Miyashita et al., 2014; Allakhverdiev et al., 2016; Ho et al., 2017c; Nürnberg et al., 2018; Kurashov et al., 2019). FaRLiP is an acclimation response that allows some cyanobacteria to utilize FRL for photosynthesis and growth. This response includes the biosynthesis of both Chl *d* and Chl *f* (Gan et al., 2014, 2015), and it is accompanied by extensive remodeling of PSI, PSII, and the PBS (Airs et al., 2014; Gan et al., 2014, 2015; Gan and Bryant, 2015; Ho et al., 2016, 2017a,b,c; Bryant and Canniffe, 2018; Ho, 2018). More than 15 species, which are distributed throughout the full taxonomic diversity of cyanobacteria, contain the FaRLiP gene cluster, and FaRLiP has been experimentally demonstrated in a growing number of cyanobacteria (Airs et al., 2014; Gan et al., 2014, 2015; Miyashita et al., 2014; Behrendt et al., 2015; Hirose et al., 2016; Li et al., 2016; Ho et al., 2017b; Ohkubo and Miyashita, 2017; Averina et al., 2018; Gómez-Lojero et al., 2018). FaRLiP is especially important for terrestrial cyanobacteria because they frequently occur in niches where visible light is strongly filtered by Chl *a* or strongly scattered, leading to enrichment in wavelengths longer than 700 nm. These environments include shaded areas under plant canopies, soil crusts, microbial mats, dense algal blooms, caves, beach rocks and stromatolites (Chen et al., 2012; Behrendt et al., 2015; Gan and Bryant, 2015; Trampe and Kühl, 2016; Ohkubo and Miyashita, 2017).

Cyanobacteria that perform FaRLiP contain a conserved 20-gene cluster, which also includes three regulatory genes: *rfpA*, *rfpB*, and *rfpC* (Gan et al., 2014; Zhao et al., 2015; Ho et al., 2017a,c). RfpA is a knotless phytochrome with a histidine kinase domain that is sensitive to red light and FRL. RfpB is a response regulator and transcriptional activator with two CheY domains and a DNA binding domain. RfpC is a small response regulator with a CheY domain, which likely acts as a phosphate shuttle between RfpA and RfpB (Gan et al., 2014; Zhao et al., 2015). RfpA senses FRL and activates RfpC and RfpB sequentially, most likely through phosphorylation; RfpB then activates transcription of the FaRLiP gene cluster (for convenience, these three related proteins/genes will hereafter be referred to collectively as RfpABC/*rfpABC*) (Zhao et al., 2015; Ho et al., 2017a,c). Although it is not yet known whether phosphorylation or dephosphorylation activates the RfpABC regulatory cascade, the well-characterized mechanism in the case of plant phytochromes involves signaling to downstream response regulators through autophosphorylation and phosphotransfer (Rockwell et al., 2006). When the *rfpABC* genes were individually deleted in *Chlorogloeopsis fritschii* PCC 9212, *Chroococcidiopsis thermalis* PCC 7203, or *Synechococcus* sp. PCC 7335, the resulting mutants were no longer able to synthesize Chl *f*, nor did they accumulate detectable levels of the subunits of PSI, PSII, and PBPs characteristically produced from the FaRLiP gene cluster in cells grown in FRL (Zhao et al., 2015; Ho et al., 2017a,c).

Chl *d* only represents about 1% of total Chl produced by cells grown in FRL (Airs et al., 2014; Gan et al., 2014, 2015; Ho et al., 2016; Ho, 2018). Recent studies indicate that Chl *d* is not a component of FRL-PSI complexes (Ho, 2018; Li et al., 2018; Nürnberg et al., 2018; Kurashov et al., 2019), and one molecule of Chl *d* may occur as an essential component of the electron transfer chain in FRL-PSII complexes (Nürnberg et al., 2018). Surprisingly, the synthesis of Chl *d* seemed to be regulated differently from these other FaRLiP processes (Zhao et al., 2015), and a first objective of this study was to clarify this unexpected result. Chl *f* synthase (ChlF) catalyzes Chl *f* synthesis in a light-dependent manner, and it was recently shown to be a paralog of PsbA, a core PSII subunit (Ho et al., 2016; Shen et al., 2019). However, in spite of several studies, the biosynthetic reaction(s) leading from Chl *a* to Chl *d*, one of the last uncharacterized steps in Chl biosynthesis, is still unknown (Schliep et al., 2010; Chen, 2014; Yoneda et al., 2016; Ho et al., 2017c). Thus, a second major objective of this study was to clarify the regulation of Chl *d* synthesis in a strain performing FaRLiP, as a means for possibly discovering the gene(s) responsible for Chl *d* synthesis. Detailed mechanistic knowledge of the biosynthesis of FRL-absorbing Chls might eventually lead to crop plants modified to have expanded photosynthetic light-wavelength utilization and ultimately higher yields by the introduction of the biosynthetic pathways for Chl *d* and/or Chl *f* into those plants (see Chen and Blankenship, 2011; Blankenship and Chen, 2013; Bryant, 2016; Ho et al., 2016).

Although RfpABC clearly regulate genes within the FaRLiP cluster as well as the overall acclimation response (Zhao et al., 2015; Ho et al., 2017b), it has remained uncertain whether RfpABC also directly regulate other physiological and metabolic responses to FRL. Thus, a third objective of this study was to determine the relationship between RfpABC and the transcription of genes leading to other responses to FRL. These three objectives are discussed below on the basis of pigment analyses and transcriptomic profiling of *rfpA*, *rfpB*, and *rfpC* mutant strains in the FaRLiP cyanobacterium, *Chlorogloeopsis fritschii* PCC 9212.

## Materials and Methods

### Strains and Growth Conditions

The wild-type (WT) strain of *Chlorogloeopsis fritschii* PCC 9212 (hereafter *C. fritschii* 9212), was obtained from the Pasteur Culture Collection^1^ (Rippka et al., 1979). The *rfpA*, *rfpB*, and *rfpC* deletion mutants of this strain were described in a previous study (Zhao et al., 2015). The growth and light conditions were similar to those previously described (Ho et al., 2016, 2017a). B-HEPES growth medium, supplemented with Em (5 or 20 μg ml^−1^) as indicated, was used for *C. fritschii* 9212 and its mutants (Dubbs and Bryant, 1991; Zhao et al., 2015). When required, erythromycin (Em) was added to media by dilution from a concentrated stock solution (40 μg ml^−1^ in ethanol). Cell growth was monitored by following changes in the optical density at 750 nm (OD_750_) as a function of time with a GENESYS 10 spectrophotometer (Thermo Spectronic, Rochester, NY, United States).

The growth temperature was ∼38°C, WL was provided by cool-white fluorescent bulbs at ∼250 μmol photons m^−2^ s^−1^, and cultures were sparged with 1% (v/v) CO_2_ in air. LED panels with emission centered at 720 nm and WL filtered by a combination of green (GamColor_660) and red (GamColor_250) plastic filters (Parlights, Inc., Frederick, MD, United States) were used to provide FRL at ∼400 μmol photons m^−2^ s^−1^ (for additional details, see Gan et al., 2014; Ho et al., 2016; Shen et al., 2019). As described by Gan et al. (2014), when these red and green filters are combined, only FRL with wavelengths greater than ∼700 nm are transmitted. The LED panels comprise 50 Epitex, L720-06AU LEDs (Marubeni, Santa Clara, CA, United States), with emission nominally centered at 720 nm. Although these LEDs nominally emit “720 nm” light, according to the manufacturer, the peak emission for individual LEDs ranges from 705 to 735 nm. Furthermore, the bandwidth characteristics for these LEDs are such that light from ∼660 to ∼760 nm is actually emitted^2^. Irradiance values for WL were measured with QSL-100 radiometer (Biospherical Instruments Inc., San Diego, CA, United States). Irradiance values for FRL were measured with an MP-200 pyranometer (Apogee Instruments, Inc., Logan, UT, United States).

### Pigment Extraction and HPLC Analysis

Cyanobacterial cells were harvested by centrifugation at 6,797 × *g* for 2 min in a table-top centrifuge at room temperature. The pelleted cells were washed once and resuspended in a minimal volume of 50 mM Tris-HCl buffer, pH 7.5. After removal of supernatant, the pellet was extracted with 270 μl of acetone:methanol (7:2) to which IAA (30 μl of a 18 mg ml^−1^ stock) was added as indicated for some experiments. Alternatively, the IAA solution was replaced by distilled water when testing the effects of this thiol-modifying reagent. The resulting solution was mixed with ∼100 μl of glass beads (150–212 μm, Sigma-Aldrich, St. Louis, MO, United States) and mounted on a bead beater; cell suspensions were agitated twice for 30 s at 4,200 rpm to extract pigments. After centrifugation at 15,294 × *g* in a table-top microcentrifuge for 3 min, the resulting supernatant was collected and subjected to RP-HPLC analysis. The HPLC analytical method has previously been described in detail (Gan et al., 2014).

### Transcription Profiling by RNAseq Analysis

Two profiling experiments were performed. In first experiment, cultures of WT *C. fritschii* 9212 and the individual *rfpABC* deletion mutants were grown in triplicate in B-HEPES medium without (WT strain) or with (*rfpABC* deletion mutants) supplementation with Em (20 μg ml^−1^) in WL to OD_750_ = ∼0.25 to 0.4. A 15-ml aliquot from each tube was collected, and the three aliquots were combined, quickly harvested by centrifugation at 4°C, rapidly frozen in liquid nitrogen, and stored at −80°C (*t* = 0 h, WL sample). The remaining samples were transferred to FRL for 2 days (*t* = 48 h, FRL sample), and cells were harvested by the same procedure. The design of the second transcription profiling experiment is illustrated schematically in Supplementary Figure S1. In this experiment, WT *C. fritschii* 9212 cultures were grown in two triplicate sets, and the individual *rfpB and rfpC* deletion mutants were grown in triplicate in B-HEPES media without supplementation with Em in WL to OD_750_ = 0.4–0.5. Aliquots (15 ml) were harvested as described above before transfer to FRL. A sublethal amount of Em (5 μg ml^−1^) was added to the remaining cells of the first set of three WT cultures, and cells were grown in WL for 48 h before cell harvest. The second set of triplicate WT cultures was transferred to FRL for 48 h. A 15-ml aliquot of cells was harvested from each culture, and then a sublethal concentration of Em (5 μg ml^−1^) was added to the remaining cultures. These cultures were grown in FRL for another 48 h. The *rfpB* and *rfpC* mutants were grown in WL without addition of Em and then transferred to FRL for 48 h before cells were harvested. RNA isolation, rRNA removal, cDNA synthesis and sequencing, and data analysis were performed as previously described (Zhao et al., 2015). The only modification of the previously described procedures was to increase the two periods of bead-beating during RNA extraction to 30 s each at 4200 rpm. Supplementary Table S1 summarizes the total read counts, mapped reads (excluding the reads mapping to rRNA), and uniquely mapped reads for the WT strain and the *rfpA*, *rfpB*, and *rfpC* deletion mutants grown in WL or FRL, with and without the presence of Em. The RNA sequencing data were deposited in the NCBI GEO database archive under accession number GSE123329.

### Analysis of Transcriptional Data

The cDNA sequences obtained from RNAseq were mapped against the *C. fritschii* 9212 genome with the Burrows-Wheeler algorithm (Li and Durbin, 2009), allowing four mismatches (>90% sequence identity). All sequences that mapped to regions coding for ribosomal RNAs and tRNAs, and reads that did not map to unique positions, were removed from the resulting mapped sequence files. Protein-coding regions were analyzed for cDNA sequences mapping entirely within or partially covering the respective ORF (overlapping by at least one nucleotide), and the resulting hits for each ORF were counted (Ludwig and Bryant, 2011). It should be noted that the *rfpA*, *rfpB*, and *rfpC* mutants do not completely delete the coding sequences of the respective genes (see Zhao et al., 2015), and the mapping algorithm can still detect partial transcripts for this reason. The relative transcript abundances for all ORFs were calculated as the number of sequences mapping in a given ORF divided by the total number of sequences mapping within any protein-coding region. To monitor differences in transcript levels between two conditions, the relative transcript abundances under the two conditions were compared for all ORFs. Ratios are tabulated as the relative transcript abundance under condition 2 divided by the relative transcript abundance under condition 1. Whenever “standard” conditions with multiple data sets served as the basis for a comparison, the probability for equal transcription was calculated for each ORF using the z-test. When data from cells grown under specialized conditions were compared, for which only single datasets were available, the chi-square test was applied to determine the probability level for equal transcription for each ORF.

To compare the mRNA levels for different genes within one sample, the number of aligned sequences for a given ORF was normalized by the length of the ORF, and the results are reported as aligned sequences (“hits”) per kilobase. For comparisons of the same gene but in different samples, the number of aligned sequences was normalized relative to the total number of mRNA counts, because the gene length is constant but the total number of mRNA counts was variable and was also dependent upon the sequencing depth.

## Results and Discussion

### Transcriptional Profiling of *rfpA*, *rfpB*, and *rfpC* Mutants in *C. fritschii* PCC 9212 Yields Insights Into the Regulation of Chl *d* Synthesis in FRL

**FIGURE 1:**
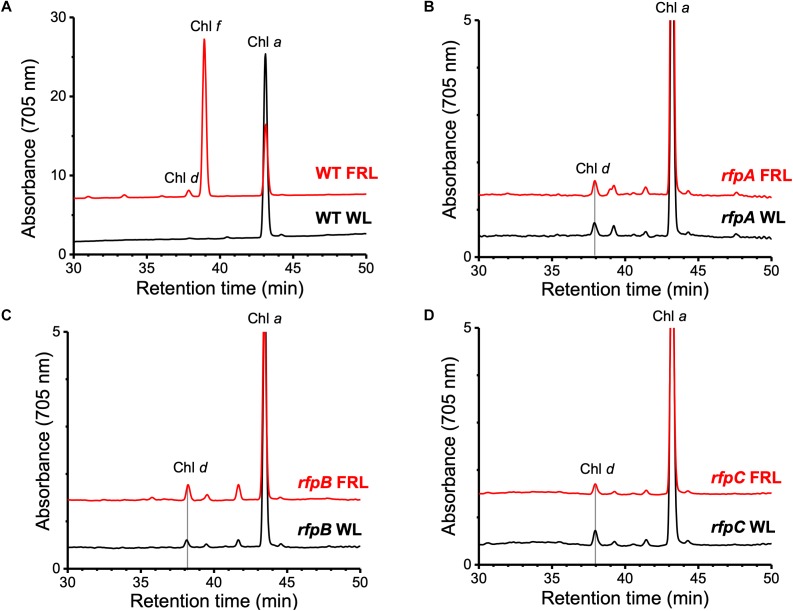
Elution profiles at 705 nm for HPLC analyses of pigments from cells of WT, *rfpA*, *rfpB*, and *rfpC* strains of *C. fritschii* 9212 grown in WL or FRL. The growth conditions are described in the Section “Materials and Methods.” **(A)** WT; **(B)** *rfpA* mutant; **(C)** *rfpB* mutant; and **(D)** *rfpC* mutant. Retention times for Chl *a*, Chl *d*, and Chl *f* are indicated. Elution profiles for extracts from cells grown in FRL were arbitrarily displaced from those for the WL control cells, and the *y*-axis was expanded *in silico* in **(B–D)** to facilitate comparison. Minor peaks eluting between 39 and 42 min in the samples from mutants had absorption spectra similar to Chl *a* and may represent molecules with esterifying alcohols other than phytol.

Figure 1A shows the RP-HPLC elution profiles for Chl pigments extracted from WT *C. fritschii* 9212 cells grown in WL or FRL. Cells grown in WL only synthesize Chl *a*, while WT cells grown in FRL synthesize Chls *a*, *d*, and *f* (Airs et al., 2014; Gan et al., 2014, 2015; Zhao et al., 2015; Ho et al., 2017a). When cells were grown in FRL for 48 h in the presence of Em, no Chl *f* synthesis was detected in the *rfpA*, *rfpB*, and *rfpC* deletion mutants (Figure 1B–D); previous studies additionally showed that such cells no longer accumulate the paralogous PSI, PSII, and PBS complexes typically observed in WT cells grown in FRL (Zhao et al., 2015; Ho et al., 2017a). Chl *d* was still detected in pigment extracts from cells of each *rfp* mutant grown in FRL. Unexpectedly, Chl *d* was also present in pigment extracts from each *rfp* mutant of *C. fritschii* 9212 when cells were grown in WL in the presence of Em (Figure 1). Assuming that the synthesis of Chl *d* is actually dependent upon an enzymatic activity, these data are *prima facie* evidence that gene(s) required for the synthesis of Chl *d* are constitutively expressed when any of the three *rfp* transcriptional regulatory genes are mutated.

We reasoned that it might be possible to identify candidate genes involved in Chl *d* synthesis by comparing global transcription patterns obtained by RNAseq for WT and the *rfpABC* mutant strains grown in WL or FRL. Previous transcriptomic analyses had demonstrated that, within 48 h of transfer of cells to FRL, transcript levels for genes of the FaRLiP gene cluster increase quite dramatically (up to 5,900-fold) in *C. fritschii* 9212 cells (Zhao et al., 2015). Under similar conditions, transcript levels for the genes of the FaRLiP cluster increased much more modestly (only up to sevenfold) in *Synechococcus* 7335 (Ho et al., 2017a). Results from other physiological and biochemical analyses were consistent with the transcription results and suggested that *C. fritschii* 9212 acclimates to FRL much more rapidly and dramatically at the transcriptional level than *Synechococcus* 7335 (Zhao et al., 2015; Ho et al., 2016, 2017a,b). Thus, WT and *rfpABC* mutant strains of *C. fritschii* 9212 were chosen for detailed transcriptional profiling experiments by RNAseq.

**FIGURE 2:**
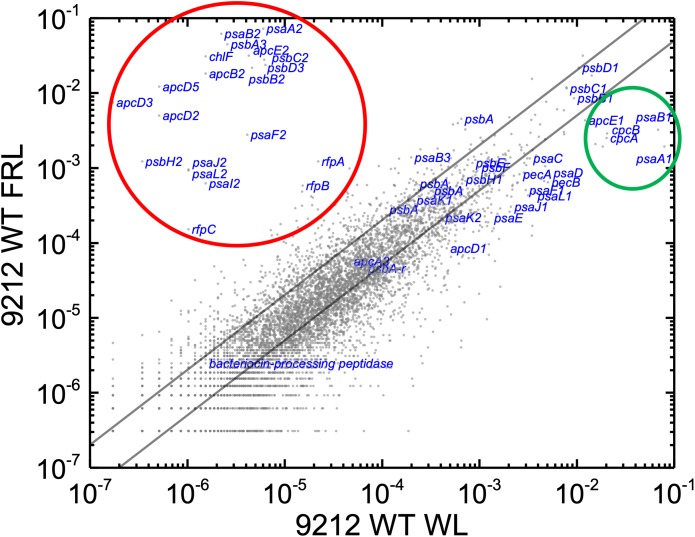
Scatter plot showing a comparison of relative transcript abundances for *C. fritschii* 9212 cells grown in FRL compared to WL. Scatter plot of RNAseq data comparing relative transcript abundance for WT *C. fritschii* PCC 9212 cells grown in FRL for 48 h (9212 WT FRL) and WL (9212 WT WL). Each gray dot represents one gene. The *X*- and *Y*-axes represent normalized transcript abundances for each condition. Genes of interest, including genes in FaRLiP gene cluster (red circle) and genes encoding PSI, PSII, and PBP/PBS subunits are labeled in blue font. The green circle shows some genes, including *apcE1*, *cpcBA*, and *psaA1B1* that are normally highly expressed in WL but have much lower relative transcript abundances in FRL. The diagonal lines indicate the thresholds for a twofold increase and a 50% reduction in relative transcript abundance, respectively.

In order to know the physiological state of the cells employed for all analyses precisely, the same cultures employed for pigment analysis in Figure 1 were used for RNAseq analysis. Cells were grown to mid-exponential phase in WL, and aliquots of the WT and *rfpA, rfpB*, and *rfpC* mutant cultures were collected and pooled from triplicate cultures as WL control samples. The remaining cells were transferred to FRL for 48 h prior to RNA extraction, rRNA depletion, and RNAseq analysis. A comparison of results for WT cells grown in WL and FRL produced results that were consistent with those from previous studies (Gan et al., 2014; Zhao et al., 2015); this experiment showed the reproducibility of this approach (data not shown). Cells rapidly acclimated to growth under FRL at the transcriptional level (Figure 2). Relative transcript levels for the 20 genes of the FaRLiP gene cluster increased dramatically in WT cells grown in FRL for 48 h (Figure 2, red circle). Supplementary Table S2 shows the normalized relative transcript levels for each gene in the WT and each of the three *rfp* mutant strains of *C. fritschii* 9212 under these two light conditions. Relative transcript abundances for the paralogous PBP and PSI genes normally expressed in WL generally decreased by 50% or more in cells grown in FRL (see examples in the green circle); however, transcript levels for most PSII genes, other than those encoding proteins specifically replaced during FaRLiP (Gan et al., 2014), remained more or less constant in cells grown in FRL for 48 h (Figure 2; see Supplementary Table S2).

**FIGURE 3:**
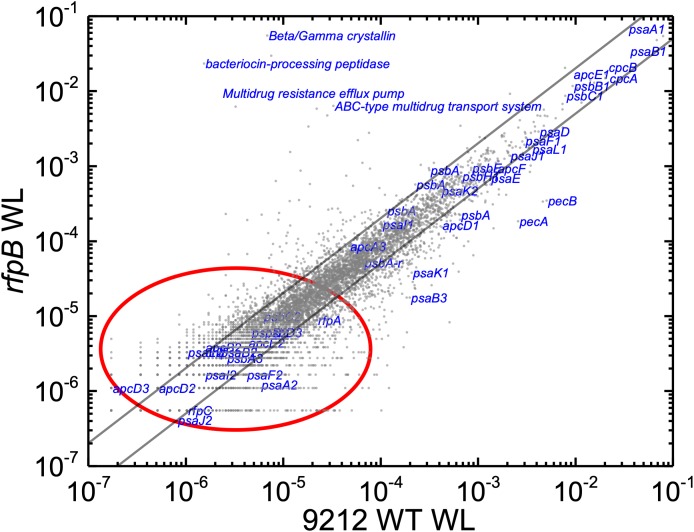
Scatter plot showing a comparison of relative transcript abundances in cells of the *rfpB* mutant and WT strains of *C. fritschii* 9212 grown in WL. The *X*- and *Y*-axes represent normalized transcript abundances in the *rfpB* mutant and the WT grown in WL, respectively. Each gray dot represents one gene. Genes of interest, including genes in FaRLiP gene cluster (red oval) and genes encoding PSI, PSII, and PBP/PBS subunits are labeled in blue. The diagonal lines indicate the thresholds for a twofold increase and a 50% reduction in relative transcript abundance, respectively.

Based upon the results described above for Figure 1, one can hypothesize that, compared to WT *C. fritschii* 9212 cells grown in WL, transcript levels for genes related to Chl *d* synthesis should be elevated in cells of all three *rfp* mutants whether grown in WL or FRL. Figure 3 shows a representative scatter plot showing the relative transcript abundances for the *rfpB* mutant of *C. fritschii* 9212 grown in WL (Chl *d* synthesized) compared to transcript abundances for the WT strain grown in WL (no Chl *d* synthesized). Similar plots are shown for the *rfpA* and *rfpC* mutant strains in Supplementary Figure S2. These comparisons showed that transcript levels for several genes increase dramatically in the *rfp* mutants grown in WL (up to 15,000-fold) compared to WT cells (Figure 2, 3 and Supplementary Table S2). In each case, the gene showing the largest increase in transcript abundance in the mutants is annotated as a beta/gamma crystallin (UYEDRAFT_05690). Other genes showing large increases in transcript abundance in the *rfpABC* mutant cells grown in WL putatively encode a bacteriocin-processing peptidase (UYEDRAFT_05692), a multidrug-resistance efflux pump (UYEDRAFT_05693), and a subunit of an ABC-type multidrug transporter (UYEDRAFT_04486) (Figure 3 and Supplementary Figure S2). The latter two genes are probably involved in antibiotic resistance. The *rfpABC* mutants are resistant to the antibiotic Em, because the coding regions of each *rfpABC* gene was replaced with a DNA fragment encoding *ermC*, which encodes an rRNA adenine *N*-6 methyltransferase that confers resistance to the macrolide antibiotic, Em (Zhao et al., 2015). The *rfpABC* mutants are routinely grown in the presence of Em, but it is possible that, in spite of their resistance to Em, these cells nevertheless sense this xenobiotic compound in the growth medium and increase the relative transcript levels of genes that contribute to resistance. It should be noted that by comparing WT and *rfpABC* mutants grown in WL, relative transcript levels for other genes (924 in *rfpA*, 659 in *rfpB*, and 512 in *rfpC* mutants, respectively) also increase above the two-fold threshold in the mutants; therefore, we arbitrarily designated these genes as demonstrating relevant changes in relative transcript levels.

**FIGURE 4:**
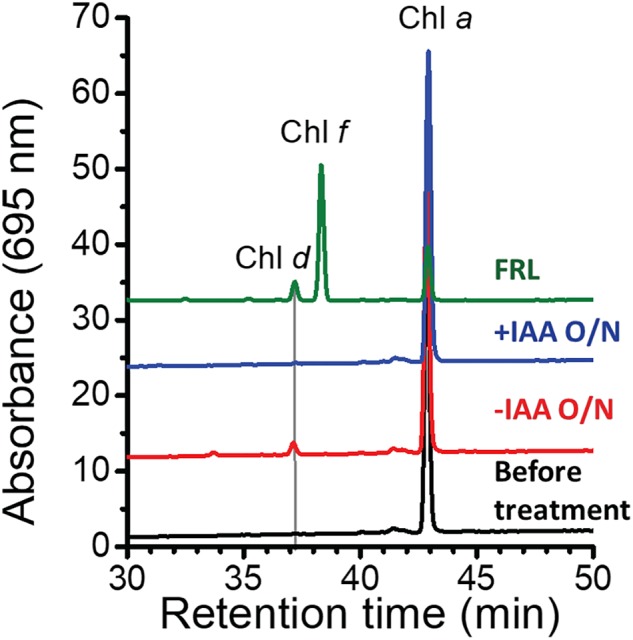
Effects of thiol compounds on Chl *d* production in pigment extracts from *C. fritschii* 9212. The figure shows the RP-HPLC elution profiles of pigments at 695 nm, the absorption maximum of Chl *d*. Pigments were extracted from *C. fritschii* 9212 cells grown in WL or FRL (green line, control). RP-HPLC analysis was then performed before treatment (black line), after incubation at room temperature overnight in the dark without IAA (–IAA O/N; red line) or after IAA treatment (+IAA O/N; blue line). The retention times of Chl *d*, Chl *f*, and Chl *a* are indicated. Pigments extracted from cells grown in FRL show the retention times for Chl *d*, Chl *f*, and Chl *a*, respectively. IAA, iodoacetamide; O/N, overnight.

At first sight, the genes showing the greatest increases in relative transcript abundance in the mutants producing Chl *d* do not appear to be related to Chl *d* biosynthesis. However, one of the genes, the bacteriocin-processing peptidase, is predicted to be a cysteine protease with an active-site thiol group. Previous *in vitro* studies showed that another cysteine protease, papain (papaya proteinase I), promotes Chl *d* formation from Chl *a* (Koizumi et al., 2005). The mechanism of Chl *d* production was further explored in subsequent studies that showed that thiolphenol can promote the conversion of Chl *a* into Chl *d* (Fukusumi et al., 2012; Loughlin et al., 2014). This prompted us to test whether thiol compounds (e.g., glutathione) or proteins with thiol groups might promote Chl *d* formation in pigment extracts (Figure 4). IAA is a powerful inhibitor of thiol compounds and enzymes with active-site cysteines because it irreversibly alkylates sulfhydryl groups (Smythe, 1936). We prepared a pigment extract from *C. fritschii* 9212 cells grown in WL, and only Chl *a* was detected in the freshly prepared pigment extract (Figure 4). When this extract was incubated overnight in the dark at room temperature under oxic conditions, some Chl *d* was formed (Figure 4, −IAA O/N). However, if IAA was added to the same extract and otherwise incubated under the same conditions, no Chl *d* was detected (Figure 4, +IAA O/N). Collectively, these results imply that thiol compounds or proteins with thiol groups could play a role in Chl *d* synthesis in FRL *in vivo*.

### Erythromycin Induces Chl *d* Synthesis in *C. fritschii* 9212

**FIGURE 5:**
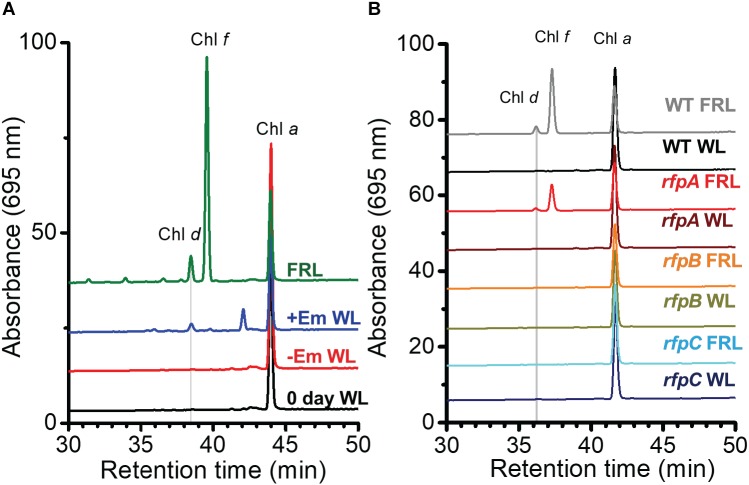
RP-HPLC elution profiles showing that erythromycin (Em) induces Chl *d* formation in *C. fritschii* 9212. Pigments were extracted and separated by RP-HPLC with wavelength monitoring at 695 nm as shown. Pigments extracted from WT cells acclimated to FRL were used as standards for Chl *a*, Chl *d*, and Chl *f* as indicated on the figure panels. Each trace represents a specific culture condition or strain. **(A)** Pigments were extracted from *C. fritschii* 9212 WT cells grown under four conditions: WL at time 0 (0 day WL; black line); cells grown in WL without Em (–Em WL; red line) for 4 days; cells grown in WL with 5 μg Em ml^−1^ for 4 days (+Em WL; blue line); and cells fully acclimated to FRL for more than 1 month (FRL; green line). **(B)** Pigments were extracted from WT and *rfpA*, *rfpB*, and *rfpC* mutant cells of *C. fritschii* 9212 that were grown in WL and transferred to FRL for 6 days. All cells were grown without addition of Em to the medium. The genotype of each mutant was verified by PCR analyses (see Supplementary Figure S4). For additional details, see text.

We further inferred from these results that it is possible that the products of genes whose transcript levels increased artifactually because of the presence of Em in the growth medium could contribute to the formation of Chl *d* in cells. Thus, further experiments were performed to define the relationship between Em treatment and Chl *d* formation. *C. fritschii* 9212 WT cells were grown in WL with or without the addition of a sub-lethal concentration of Em (5 μg ml^−1^) for 4 days, and pigments were extracted from cells and immediately analyzed by RP-HPLC. Surprisingly, the results showed that Chl *d* synthesis (but not Chl *f* synthesis) occurred in cells incubated with Em (Figure 5A, +Em WL). This result confirmed that the presence of Em in the growth medium could promote the formation of Chl *d* in WT cells of *C. fritschii* 9212 grown in WL *in vivo*. Because an *ermC* resistance cassette was used in their construction, and the resulting *rfpABC* mutants had been grown in B-HEPES medium containing Em (Zhao et al., 2015), these results invalidated all previously obtained conclusions concerning Chl *d* synthesis for the *rfpABC* mutant strains. These surprising results indicate that it may be a good practice to evaluate carefully the effects of antibiotics alone on relative transcript levels in transcription profiling experiments involving mutant strains constructed with antibiotic resistance markers.

### Chl *d* Synthesis Is Regulated by RfpABC in *C. fritschii* 9212

To determine whether RfpABC are truly involved in regulating the synthesis of Chl *d*, each *rfp* mutant strain was grown in WL or FRL in the absence of Em. As shown in Figure 5B, none of the three mutant strains synthesized Chl *d* when grown in WL in the absence of Em. This indicates that the previous detection of Chl *d* in the *rfpABC* mutants grown in WL was not due to the *rfpABC* mutations but was actually caused by the presence of Em in the growth medium (Figure 1; also see Zhao et al., 2015). More importantly, no Chl *d* synthesis occurred when the *rfpB* and *rfpC* mutants were grown in FRL in the absence of Em (Figure 5B). These results establish that, like the synthesis of Chl *f* and the expression of other genes of the FaRLiP cluster, Chl *d* synthesis requires (i.e., is potentiated by) RfpB and RfpC. This conclusion concerning the Rfp regulators is further supported by results for a *chlF* mutant that lacks Chl *f* synthase (Ho et al., 2016). The *chlF* mutant retains RfpABC but is unable to synthesize Chl *f* in FRL; however, this strain is still able to synthesize Chl *d* in the absence of Em when cells are grown in FRL (Supplementary Figure S3). Thus, neither ChlF nor Chl *f* are required for the synthesis of Chl *d in vivo* in *C. fritschii* 9212, but RfpB and RfpC are required for Chl *d* synthesis in cells grown in FRL.

The results obtained for the *rfpA* mutant were more complex. Chl *d* synthesis was not detected in *rfpA* mutant when cells were grown in WL, but small amounts of Chl *d* and Chl *f* were produced in cells of this mutant grown in FRL for 6 days (Figure 5B). The levels of Chl *d* and Chl *f* produced by the *rfpA* mutant grown in FRL were much lower than those for the WT (Figure 5B). PCR experiments were performed to verify that the results obtained for each mutant strain were not due to unexpected reversion mutations or contamination with WT cells, which might have occurred more easily in the absence of antibiotic selection with Em. The PCR experiments in each case confirmed the identity of the mutant strain (Supplementary Figure S4). Thus, we concluded that FaRLiP is weakly activated when cells of the *rfpA* mutant were grown in FRL for 6 days. In previous experiments with *C. fritschii* PCC 9212, the pigment contents of the *rfpABC* mutants were analyzed after ∼2–4 days (Zhao et al., 2015). It seems likely that, in the absence of RfpA, some other FRL-dependent photoreceptor(s) can weakly activate the FaRLiP response by phosphorylation of the phosphotransferase RfpC and the transcriptional-activating response regulator, RfpB. In the complete absence of either RfpC and RfpB, no activation of gene expression can occur, and thus transcription of the FaRLiP genes and Chl *d* synthesis does not occur. This explanation is supported by the transcription profiles of the *rfpABC* mutants grown in WL and FRL (see Figure 2 and Supplementary Figure S5). Relative transcript levels for genes of the FaRLiP cluster are very high in the WT strain, increasing by up to 43,500-fold within 48 h (Figure 2 and Supplementary Table S2). This increase in transcript levels is completely abolished in cells of the *rfpB* and *rfpC* mutants grown in FRL (Supplementary Figures S5B,C, red circles). However, transcript levels for the FaRLiP gene cluster still increase slightly after 48 h for the *rfpA* mutant (Supplementary Figure S5A, red oval). These data indicate that in the absence of RfpA some other FRL-sensitive, photosensory kinase can weakly activate RfpC and RfpB; however, in the absence of these two response regulators, no expression of the FaRLiP genes occurs in *C. fritschii* 9212. The reduced expression of the FaRLiP genes (Figure 2 and Supplementary Figure S5A) is consistent with the reduced levels of Chl *d* and Chl *f* synthesized in cells of the *rfpA* mutant (Figure 5B) in comparison to the WT, when cells were grown for 6 days in FRL.

### Erythromycin Changes Transcriptional Profiles of WT and *rfpB* and *rfpC* Mutants in WL and FRL in *C. fritschii* 9212

To eliminate the effect of Em on transcript levels in the *rfpABC* mutants, a second set of transcriptomics analyses by RNA sequencing was conducted as follows. The *rfpB* and *rfpC* mutants were cultured without Em initially in WL and then transferred to FRL for 2 days. The *rfpA* mutant was excluded from this experiment because it had already been shown to express the genes within the FaRLiP gene cluster when cells were grown for an extended period in FRL (Figure 5B and Supplementary Figure S5A). The effect of Em was also tested by incubating WT cells in WL and then supplementing the medium with a sublethal Em concentration (5 μg ml^−1^) for two additional days in WL. Another triplicate set of WT cultures was transferred from WL to FRL for 2 days and then supplemented with the same sublethal concentration of Em for another 2 days in FRL (see section “Materials and Methods” and Supplementary Figure S1). PCR analyses were used to verify the absence of contamination by WT cells (or revertants) in the *rfpB* and *rfpC* mutant cultures after incubation in FRL (Supplementary Figure S6). The results are summarized in Supplementary Table S3.

**FIGURE 6:**
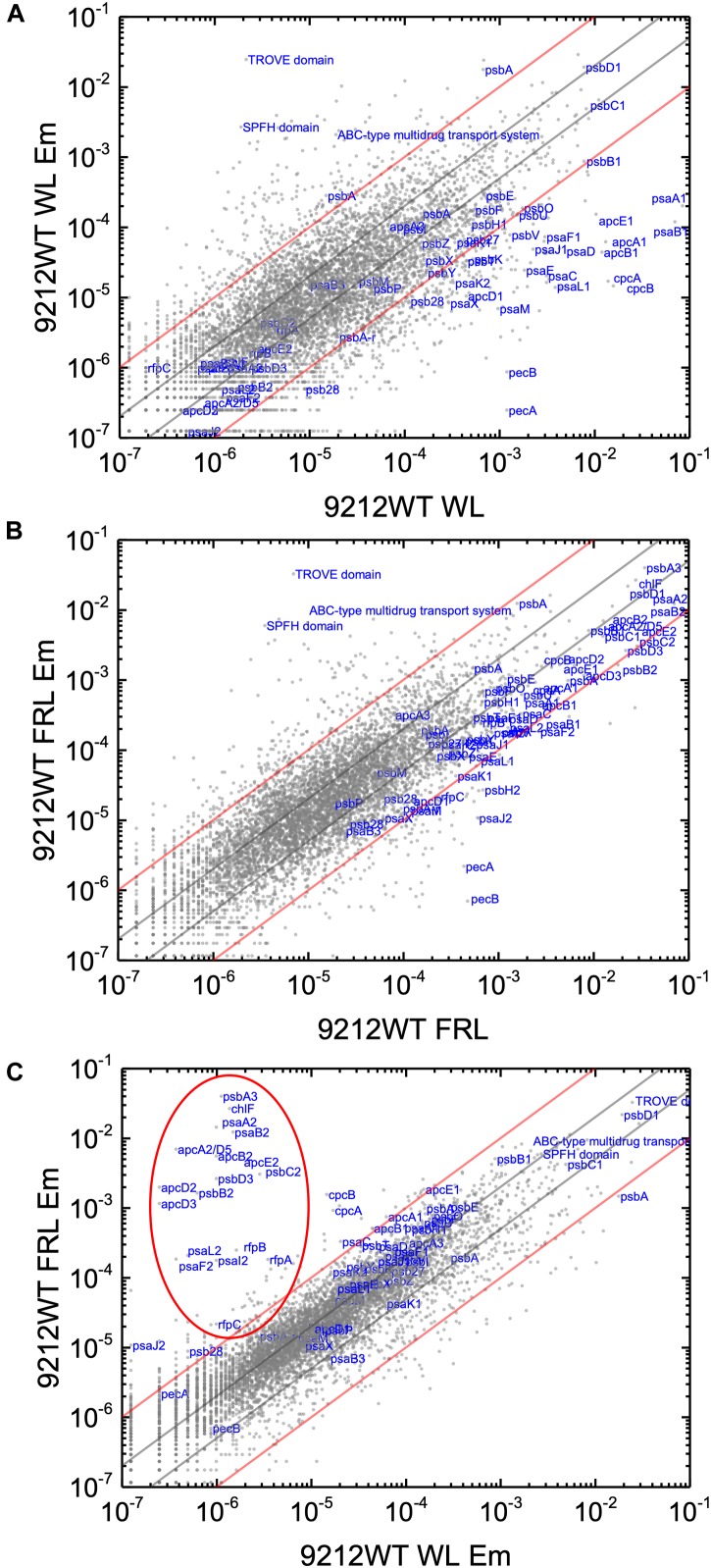
Scatter plots showing comparisons of relative transcript abundances for *C. fritschii* 9212 cells grown with or without Em in WL and FRL. Scatter plot of RNAseq data comparing relative transcript abundance for WT *C. fritschii* 9212 cells grown **(A)** in WL (9212WT WL) and supplemented with Em (5 μg ml^−1^) for 48 h in WL (9212WT WL Em) and **(B)** in FRL (9212 WT FRL) for 48 h and supplemented with Em (5 μg ml^−1^) for another 48 h in FRL (9212WT FRL Em). An additional comparison of relative transcript abundances for (9212WT WL Em) and (9212WT FRL Em) is shown in **(C)**. Each gray dot represents one gene. The *X*- and *Y*-axes represent normalized transcript abundances for each condition. Genes of interest, including genes in FaRLiP gene cluster (in the red oval, except *psaJ2*), and genes encoding PSI, PSII, and PBP/PBS subunits are labeled in blue font. Genes in the FaRLiP gene cluster are not circled because relative transcript abundances of these genes are not different from those of most of other genes under the conditions shown in **(A,B)**. The gray and red diagonals indicate the thresholds for a 2-fold and 10-fold increase and a 50 and 10% reduction in relative transcript abundance, respectively.

The relative transcript abundances in the WT strain grown in WL and FRL show very similar trends in the second set of samples as in the first set, which indicates that the experimental conditions were consistent and that the results are highly reproducible (e.g., compare Figure 2 and Supplementary Figure S7). However, addition of a sublethal concentration of Em affected the relative transcript abundances of many genes in the WT strain (Figure 6). For example, in WT cells grown in WL, the addition of Em caused a dramatic decrease of transcript levels of PSI and PBS related-genes. Most decreased more than 90–99%, and the highest decrease was 99.98% for *pecA*, which encodes the α subunit of phycoerythrocyanin, (Figure 6A and Supplementary Table S3). In WT cells grown in FRL, Em also caused an approximate 90–95% decrease of the relative transcript levels of genes in FaRLiP cluster; however, relative transcript levels of those genes are still higher than for cells grown in WL (Figure 6B,C), but the highest decrease (99.99%) occurred for a gene outside of FaRLiP gene cluster, *pecB*, which encodes the β subunit of phycoerythrocyanin (Figure 6B and Supplementary Table S3). For unknown reasons, transcript abundances of genes encoding a TROVE domain protein (UYEDRAFT_04043) and an SPFH domain protein (UYEDRAFT_02422), increased dramatically in WT cells grown in both WL and FRL after addition of Em (Figure 6 and Supplementary Table S3). The relative transcript abundance of a gene encoding a component of an ABC-type multidrug transport system (UYEDRAFT_04486) also increased up to 670-fold in response to Em addition (Figure 6 and Supplementary Table S3). Besides these three genes, transcript levels of many other genes increased or decreased more than 2-fold (50% decrease) or even 10-fold (90% decrease). These results indicate that a sub-lethal concentration of Em influences relative transcript abundances very significantly in this cyanobacterium.

**FIGURE 7:**
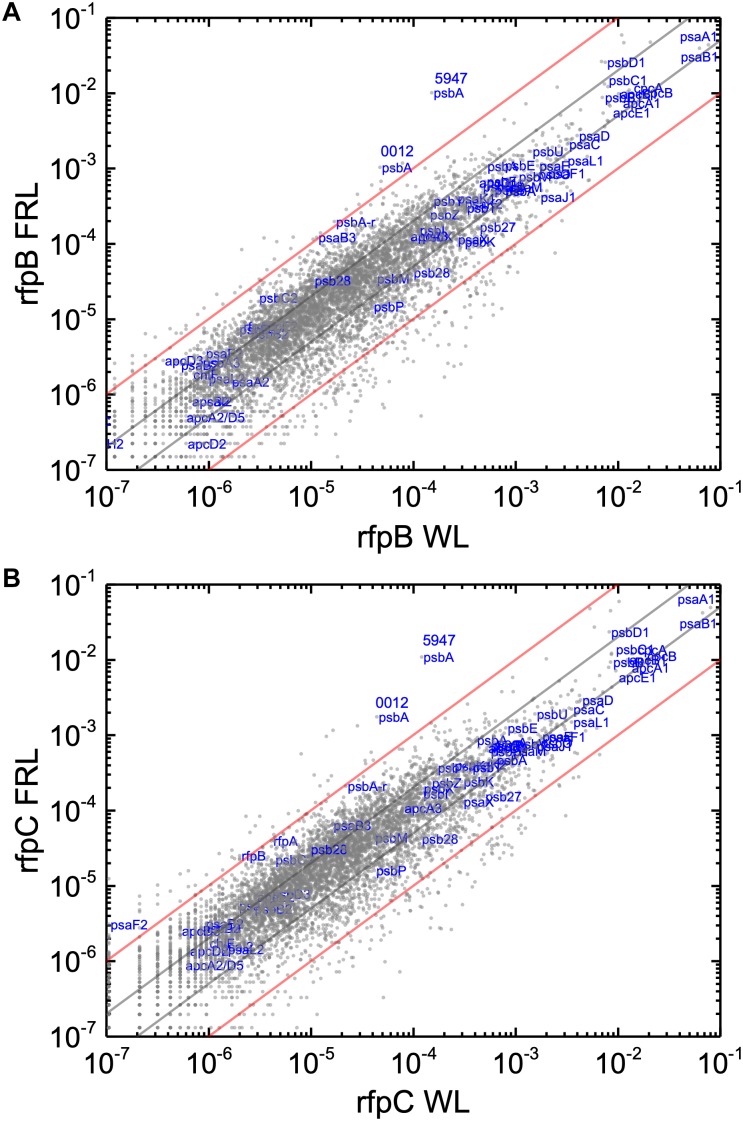
Scatter plots showing a comparison of relative transcript abundances for *C. fritschii* 9212 cells of the *rfpB and rfpC* mutants and WT grown in FRL and WL. The *X*- and *Y*-axes represent normalized transcript abundances in the **(A)** *rfpB* mutant and **(B)** *rfpC* mutant grown in FRL and in WL, respectively. Each gray dot represents one gene. Genes of interest, including genes in FaRLiP gene cluster and genes encoding PSI, PSII, and PBP/PBS subunits are labeled in blue. Two *psbA* genes (UYEDRAFT_00012 and UYEDRAFT_05947) are labeled as 0012 and 5947, respectively. The gray and red diagonals indicate the thresholds for a 2-fold and 10-fold increase and a 50 and 10% reduction in relative transcript abundance, respectively.

Without the superimposed effects caused by Em, a comparison of the relative transcript abundances between WL and FRL in the *rfpB* and *rfpC* mutants showed that relative transcript abundances for only a few genes increased by more than 10-fold or decreased by more than 90%. Furthermore, the relative transcript levels of genes in FaRLiP gene cluster were similar in cells whether they were grown in WL and FRL (Figure 7). This was anticipated because of the inability of FRL to activate the transcription of these genes in the *rfpB* and *rfpC* mutants.

The relative transcript levels in the *rfpB* and *rfpC* mutants grown in WL in the absence of Em were more consistent with those of the WT in WL (Supplementary Figure S8). Relative transcript levels for most genes fell within the twofold threshold limits (an increase of less than twofold or a decrease of less than 50%). Transcript levels for genes related to drug resistance did not increase as occurred when Em was present in the growth medium for the *rfpB* and *rfpC* mutants (Figure 3 and Supplementary Figures S2B, S8). Additionally, the relative transcript abundances for *pecA* and *pecB* did not decrease in the mutants (Figure 3 and Supplementary Figures S2B, S8). These differences indicate that Em significantly affects relative transcript levels for many genes in the *rfpB* and *rfpC* mutants even when cells have an antibiotic resistance cassette and are able to grow under selective conditions. In the absence of Em, much simpler background transcript levels were obtained, and this facilitated comparisons between the WT and mutant strains.

### Chl *d* Synthase Is Likely Encoded Within the FaRLiP Gene Cluster

**FIGURE 8:**
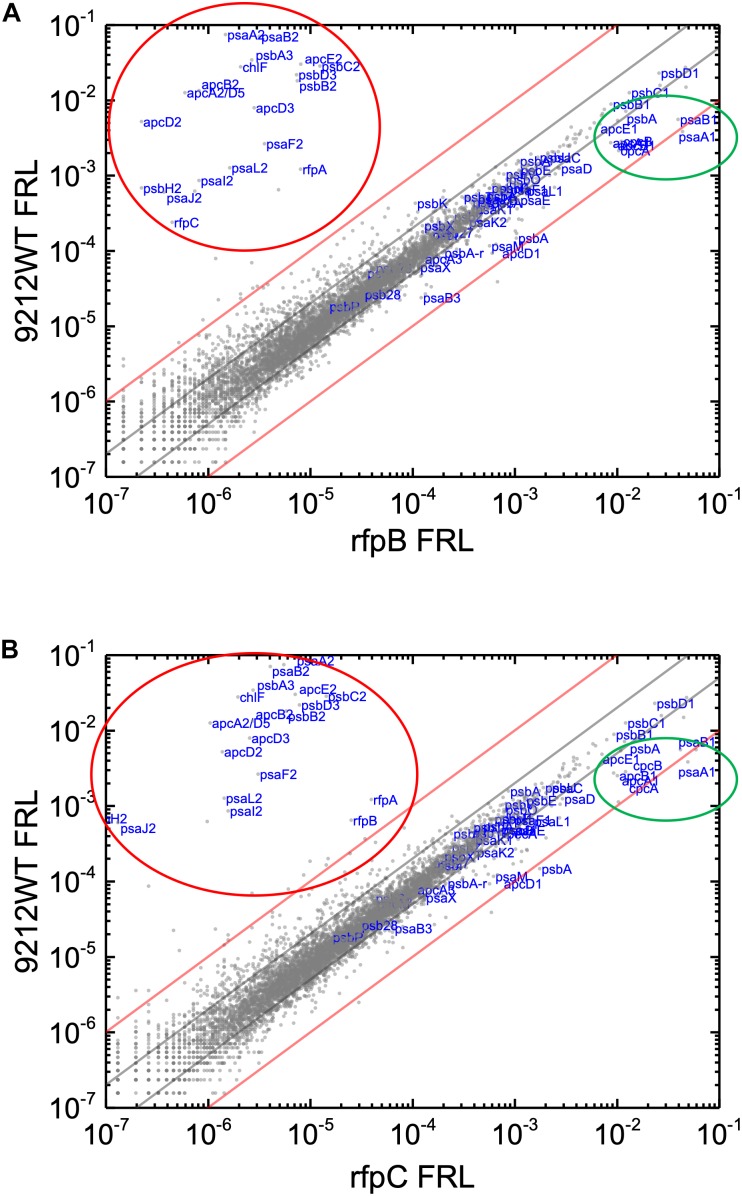
Scatter plot showing a comparison of relative transcript abundances for *C. fritschii* 9212 cells of the WT and *rfpB* and *rfpC* mutants grown in FRL. The *X*- and *Y*-axes represent normalized transcript abundances in the WT and **(A)** *rfpB* mutant or **(B)** *rfpC* mutant grown in FRL, respectively. Each gray dot represents one gene. Genes of interest, including genes in FaRLiP gene cluster (red oval) and genes encoding PSI, PSII, and PBP/PBS subunits are labeled in blue font. The green circle shows some genes highly expressed in WT in WL, including *apcE1*, *cpcBA*, and *psaA1B1*. The gray and red diagonals indicate the thresholds for a 2-fold and 10-fold increase and a 50 and 10% reduction in relative transcript abundance, respectively.

Knowing that the results without Em had a low background variability and were likely free from artifacts, appropriate comparisons should now provide a good opportunity to identify candidate genes that might encode the putative Chl *d* synthase. However, as shown in Figure 8, comparisons of the relative transcript levels between *rfpB* and *rfpC* mutants grown in FRL and WT in grown in FRL show that relative transcript levels only change dramatically for the genes encoded within the FaRLiP gene cluster. Very few other genes have comparable increases in relative transcript abundances, with nearly all increasing between two- and ten-fold. However, as shown above, Chl *d* synthesis is clearly controlled by RfpABC like other FaRLiP responses (Figure 5B). An additional search criterion is that the gene for Chl *d* synthase should be present in all FaRLiP strains but absent from all other cyanobacterial strains, with the possible exception of *A. marina* that also produces Chl *d* (Miyashita et al., 1996). A manual survey of genes upregulated fivefold or greater showed that there are no genes other than those in the FaRLiP gene cluster that fulfill both of these criteria (data not shown). From an evolutionary perspective, the FaRLiP gene cluster is distributed across five taxonomic sections of distantly related cyanobacteria, which suggests that this gene cluster has been spread through horizontal gene transfer (Gan et al., 2014; Gan and Bryant, 2015). Recently, Nürnberg et al. (2018) suggested that Chl *d* might be a component of the electron transfer chain in PSII, which potentially would cause Chl *d* to be essential for growth in FRL. One way to ensure that the capacity to synthesize a potentially essential component of PSII in FRL would be to encode the capacity for its synthesis within the FaRLiP gene cluster. Although *chlF* was once annotated as *psbA4*, a paralog of the PSII gene, *psbA*, it turned out that ChlF catalyzes Chl *f* synthesis rather than encoding a PSII subunit involved in water oxidation (Ho et al., 2016). These considerations lead to the surprising but nearly inescapable hypothesis that Chl *d* synthase, like Chl *f* synthase, must be encoded within the FaRLiP gene cluster and that it is a divergent paralog of a protein that has gained a new function.

### Transcriptional Responses in FRL of Gene for PSI, PSII, and PBS Subunits Encoded Outside the FaRLiP Gene Cluster

In addition to the FaRLiP response, other physiological and metabolic changes occur when cells are shifted from WL into FRL (Gan et al., 2014; Gan and Bryant, 2015). Transcript levels for ∼2,661 genes (∼40% of the genome) increased by more than twofold or decreased by more than 50% when WT *C. fritschii* 9212 cells were shifted from WL to FRL for 48 h (Supplementary Figure S7 and Supplementary Table S3). This is similar to changes in relative transcript levels that occur when *Leptolyngbya* sp. JSC-1 cells are shifted from WL to FRL. In that organism relative transcript levels for ∼2,900 genes (∼40% of the genome) increase at least twofold or decrease by 50% or more upon a shift from WL to FRL (Gan et al., 2014). As discussed above, RfpABC are important transcriptional regulators that control transcription of the FaRLiP gene cluster and, as now shown here, the gene(s) for Chl *d* biosynthesis. However, it was unknown whether RfpABC also control transcriptional responses producing other physiological and metabolic responses to growth in FRL. Because transcriptomic analyses of the WT and *rfpA*, *rfpB*, and *rfpC* mutants were performed in this study, it should be possible to determine whether RfpABC regulate other processes by comparing transcript levels in WT and mutant cells shifted from WL and FRL.

Relative transcript levels for genes of the FaRLiP gene cluster are extremely low in the *rfpB*, and *rfpC* mutants whether cells are grown in WL or in FRL, but there are still a few genes outside the twofold/50% threshold limits (Figure 7 and Supplementary Figure S8). These other genes outside the FaRLiP gene cluster that are related to light harvesting and light-energy conversion are clearly regulated differently (Figure 7 and Supplementary Tables S3, S4). Relative transcript levels for genes encoding PBP and PSI that are not encoded in the FaRLiP gene cluster are generally more abundant in cells grown in WL and are substantially reduced in cells grown in FRL. For example, relative transcript levels for the *apcA1B1* and *cpcBA* genes in cells grown in FRL are 80–90% lower than the levels observed in WT cells grown in WL (Figure 2 and Supplementary Figure S7). Notable exceptions are the *nblA1* and *nblA2* genes, for which transcript levels increase two–threefold in FRL. An increase in NblA should promote the degradation of PBP by forming a ternary complex comprising ClpC (the HSP100 partner of Clp proteases), NblA, and a PBP subunit (Karradt et al., 2008; Baier et al., 2014). These transcription patterns are fully consistent with the generally lower PBP levels observed in cells that have acclimated to FRL (Chen et al., 2012; Gan et al., 2014; Li et al., 2014; Zhao et al., 2015; Ho et al., 2017a). Notably, transcript levels for many PBS- and PBP-related genes are not as severely reduced in FRL in the *rfpBC* mutants as in the WT; transcript levels are up to 10 times higher in the *rfpBC* mutants than in the WT strain in FRL-grown cells (Figure 8 and Supplementary Tables S3, S4). This might be because the transcriptional (and translational) apparatus is “more available” in the mutants than in the WT. RNA polymerase molecules in the WT would be actively transcribing the genes of the FaRLiP cluster, which is not the case in the *rfpBC* mutants. The other possibility is that RfpABC are directly or indirectly involved in downregulation of PBS- and PBP-related genes in FRL to facilitate the remodeling of those complexes using subunits encoded in FaRLiP gene cluster.

The relative transcript levels for *psaA1B1*, which encode the core heterodimer subunits of PSI in WL-grown cells of *C. fritschii* 9212, are ∼95% lower in cells grown in FRL than the levels for these genes in cells grown in WL (Figure 2, Supplementary Figure S7, and Supplementary Table S3). Similar to the situation for PBP- and PBS-related genes, transcript levels *psaA1B1* are not as severely reduced in the *rfpABC* mutants (∼40–50% lower). In contrast, relative transcript levels for the *psaA2B2* genes are more than 56,000-fold higher when WT cells are grown in FRL than when grown in WL (Supplementary Tables S3, S4). The relative transcript abundance for *psaB3* increases, although transcript levels for most other PSI-related genes are lower in FRL-grown cells. Although the specific function of PsaB3 in PSI is still unknown (Magnuson et al., 2011; Gan et al., 2014; Zhao et al., 2015), transcript levels for *psaB3* increased about 2.5-fold when WT cells were grown in FRL, and the protein was also detected in PSI complexes formed in FRL (Gan et al., 2014).

Genes encoding most PSII subunits that are encoded outside the FaRLiP gene cluster are differently regulated in FRL than genes for subunits of PBP/PBS and PSI. In general, transcript levels for PSII genes are similar in WL and FRL (see Figure 2, Supplementary Figure S7, and Supplementary Tables S3, S4). Paralogous genes for PSII components (*psbA, psbB1, psbC1, psbD1*, and *psbH1*) that are expressed in WL are still expressed at substantial levels after 48 h in FRL. This may help to explain the observation that fluorescence emission from PSII complexes containing only Chl *a* continues to be observed, and perhaps is even enhanced, in *rfpABC* mutants that are unable to express the genes of the FaRLiP cluster (Zhao et al., 2015; Ho et al., 2017a). Relative transcript levels for two *psbA* genes increase differently in WT and *rfpBC* mutants in FRL. Relative transcript abundances for *psbA* genes, UYEDRAFT_00012 and UYEDRAFT_05947, increase 10- and 36-fold, respectively, in WT cells grown in FRL; however, the relative transcript levels for these two genes increase 22- and 67-fold in *rfpB* mutant and 40- and 90-fold in *rfpC* mutant. These data indicate that the PSII subunits encoded by these two *psbA* genes may have enhanced roles in FRL when FaRLiP is not functional, or may simply be much more highly transcribed when RNA polymerase is not so extensively transcribing the genes of the FaRLiP gene cluster (Figure 2, Supplementary Figure S7, and Supplementary Table S4).

### Transcriptional Response to FRL of Some Other Genes Related to Photosynthesis

Relative transcript levels for most genes encoding enzymes of tetrapyrrole biosynthesis are higher in cells grown in WL than in cells grown in FRL, and this is the case for both the WT as well as the *rfpABC* mutants (Supplementary Tables S3, S4). This is consistent with the observation that cellular Chl contents are lower in FRL than in WL in both *Synechococcus* 7335 and *C. fritschii* 9212 (data not shown; Ho et al., 2017a,b). Exceptions include *hemN*, *hemN*2, *acsF2*, *hemH*, and *ho2*, for which transcripts are more abundant in both the WT and *rfpABC* mutants in cells grown in FRL. Oxygen suppresses the transcription of four of these genes by inactivation of a [4Fe-4S] cluster in ChlR, a transcriptional activator for these genes, whose products have higher affinity for oxygen (Ludwig et al., 2014). These data indicate that the intracellular oxygen levels of cells grown in FRL are probably much lower than those in cells grown in WL. During the transition period when remodeling of the PS apparatus is occurring, it is probable that PSII activity is lower, and potentially respiratory activity is higher, than in cells grown in WL. Relative transcript levels for genes involved in synthesis of carotenoids and their precursors show similar trends to those for tetrapyrroles. Compared to cells grown in WL, their transcript levels are generally lower in both WT and *rfpBC* mutant cells grown in FRL (Supplementary Tables S3, S4). This is consistent with the observation that carotenoid levels in cells acclimated to FRL are similar to or lower than levels in WL-grown cells (Ho et al., 2017a).

*Chlorogloeopsis fritschii* 9212 contains a cluster of three genes that are homologous to the *apcD4-apcB3-isiX* genes found in low-light-adapted ecotypes of *Synechococcus* spp. of hot spring microbial mats (Olsen et al., 2015). In thermophilic *Synechococcus* spp. strains, these genes are also associated with a putative cyanobacteriochrome that is located adjacent to this gene cluster, and they are responsible for a different photoacclimation response, Low-Light-Photoacclimation (LoLiP) (Gan and Bryant, 2015; Nowack et al., 2015; Olsen et al., 2015; Ho et al., 2017c). Although the *isiX*, *apcB3*, and *apcD4* genes are adjacent to each other in *C. fritschii* 9212, *isiX* is transcribed in the opposite direction from *apcB3-apcD4*, which indicates that they must be expressed from different promoters. Transcript levels for *isiX* are higher than those for *apcD4* and *apcB3* in FRL, particularly in the *rfpABC* mutants, but the pattern of expression and transcript levels for these three genes in *C. fritschii* 9212 indicates that these genes are probably not specifically involved in acclimation to FRL and are not regulated by RfpABC (Supplementary Tables S3, S4).

The orange carotenoid protein (OCP) of cyanobacteria binds a ketocarotenoid, 3′-hydroxyechinenone, and OCP is thought to bind to PBS and quench energy transfer non-photochemically from PBS to the photosystems (Kerfeld et al., 2003; Kirolovsky and Kerfeld, 2013, 2016). Energy transfer can be restored through the action of the fluorescence recovery protein (FRP) (Sutter et al., 2013). It has recently been recognized that proteins homologous to the N-terminal and C-terminal domains of OCP also occur in cyanobacteria (Melnicki et al., 2016). The former proteins are known as helical carotenoid proteins (HCP) and the latter are C-terminal domain homologs (CTDH), but the functions of neither group are currently known. *C. fritschii* 9212 has single-copy genes for OCP1 (UYEDRAFT_03370), FRP (UYEDRAFT_03371), and one CTDH (UYEDRAFT_00381). Relative transcript levels of OCP1, HCP1 (UYEDRAFT_05176), and HCP4 (UYEDRAFT_00380) are generally lower in FRL in both WT and mutants (Supplementary Table S4). Like OCP1, HCP4 can dissipate energy from PBS under high light conditions (López-Igual et al., 2016). It is likely that FRL is perceived as a low-light condition for cells, so the expression of proteins involved in photoprotection might decrease in FRL for this reason.

### Balancing the Redox Potential in Thylakoid Membranes in FRL

We hypothesize that during the initial period of acclimation, when cells are transferred from WL to FRL but before full acclimation to FRL has occurred, the photosynthetic apparatus will be inefficient in harvesting FRL and might be unable to maintain the redox potential balance of the plastoquinone pool. The plastoquinone pool could be quickly oxidized because of limited oxidation of water by PSII and increased respiration (see above). As a result, relative transcript levels of genes encoding subunits of WL-PSII might initially increase in FRL as a response to the oxidized plastoquinone pool. The transcriptional regulation of PSII genes by the redox state of plastoquinone pool has been demonstrated in the chloroplast in plants (Pfannschmidt et al., 1999). When RfpABC are present, cells eventually acclimate to FRL by replacing WL-type PSI and PSII with remodeled FRL-type photosystems, which should ultimately reestablish the balance of the redox state of the plastoquinone pool. This could be the reason that the transcript abundance of two *psbA* genes (UYEDRAFT_00012 and UYEDRAFT_05947) are two–threefold higher in *rfpBC* mutant than WT cells in FRL (Supplementary Tables S3, S4). We also observed enhanced fluorescence emission from WL-type PSII in the *rfpABC* mutants (Figure 4 in Zhao et al., 2015 and Figure 5 in Ho et al., 2017a), which might result from enhanced expression of PSII genes due to the redox state of the plastoquinone pool (but not directly controlled by RfpABC).

In addition, to maintain the redox state of the plastoquinone during the initial period of acclimation to FRL, transcript levels for genes encoding the enzymes of the TCA cycle, pyruvate dehydrogenase, NADH dehydrogenase and succinate dehydrogenase (*sdhA* and *sdhB*) do not decrease much or at all in cells acclimating to FRL (Supplementary Tables S3, S5). However, genes for cytochrome oxidase and ATP synthase generally decrease in cells acclimating to FRL. Complexes for respiratory and photosynthetic electron transport are located in thylakoid membrane in cyanobacteria, and they share the same mobile electron carriers, plastoquinone and plastocyanin or cytochrome *c*_6_ (Mullineaux, 2014; Liu, 2016). NADH dehydrogenase and succinate dehydrogenase can also supply electrons to the plastoquinone pool in the dark or when the rate of photosynthetic electron transfer is low (Mullineaux, 2014; Liu, 2016). Cells might use oxidoreductases other than WL-type PSII (i.e., NADH dehydrogenase or succinate dehydrogenase) to assist in reducing plastoquinone to maintain the redox balance of the plastoquinone pool and provide electrons for metabolism (see below). These effects would be attenuated in WT but not in the *rfpABC* mutants in the long-term because WT cells can eventually remodel their photosynthetic apparatus, and both FRL-type PSI and PSII can productively utilize FRL and rebalance the redox state of the plastoquinone pool.

### Transcriptional Responses of Other Metabolic Processes to FRL

Considering the very large increases and decreases in relative transcript levels that occur for 1000’s of genes when cells are shifted from WL to FRL (Figure 2 and Supplementary Figure S7), it was of interest to examine transcription patterns for genes other than those encoding photosynthesis-related functions. General conclusions are that relative transcript levels for biosynthetic processes are lower, while transcript levels for biomolecule turnover and degradation are higher, in cells grown in FRL for 48 h. For example, in spite of the extensive remodeling of the photosynthetic apparatus that occurs, transcript levels for genes encoding ribosomal proteins are generally much lower (∼10–99%) after 48 h in FRL (Supplementary Tables S3, S5). Although relative transcript levels for genes encoding ribosomal proteins were also lower in the *rfpBC* mutants, the decreases were generally somewhat less severe when transcription of the FaRLiP gene cluster could not occur. These observations are counter-intuitive, because less translational machinery should be required in cells that are unable to acclimate to FRL and that grow more slowly as a result (Ho et al., 2017a; Gan et al., unpublished results). This is consistent with the notion that the transcriptional machinery is less occupied with the very high levels of expression of the FaRLiP gene cluster in the mutants, as noted above. Concerning the transcriptional apparatus, transcript levels for genes encoding the core subunits of RNA polymerase do not change much when WT cells of *C. fritschii* 9212 are grown in FRL for 48 h. However, relative transcript levels for two sigma factors (UYEDRAFT_00237 and UYEDRAFT_05654) increase ∼2-fold, and similar trends are observed in the *rfpBC* mutant strains (Supplementary Tables S4, S5). Transcript levels for several other genes encoding sigma factors decreased substantially (∼80–98%) in FRL. These patterns indicate that changes in gene expression upon a shift from WL to FRL could at least partly be due to changes in the pool of sigma factors available to RNA polymerase with accompanying changes in promoter selection by RNA polymerase.

The synthesis and degradation of fatty acids and lipids provide an additional example of differential regulation in WL and FRL. Relative transcript levels for genes involved in the synthesis of fatty acids and lipids are lower (∼10–60%) in WT and *rfpBC* mutant cells grown in FRL, but transcript levels of genes encoding enzymes required for β-oxidation of fatty acids (UYEDRAFT_05553 to UYEDRAFT_05556) are ∼10-fold higher in the WT after 48 h in FRL. In the *rfpBC* mutants, transcript levels for these genes were even higher (15- to 70-fold) (Supplementary Tables S3, S5). Similarly, relative transcript levels for some cellular and extracellular proteases are higher in cells grown in FRL, and this trend is also observed in the *rfpBC* mutants (Supplementary Tables S3, S5). Relative transcript levels for cyanophycin synthase (UYEDRAFT_03270) are similar in cells grown in WL or FRL, but transcripts for two cyanophycinases (UYEDRAFT_03269 and UYEDRAFT_02864) decrease slightly (Supplementary Tables S3, S5). On the other hand, transcript levels for genes encoding enzymes for the degradation of glycogen and other polysaccharides are relatively lower or unchanged in cells grown in FRL. Furthermore, in *C. fritschii* 9212 WT and the *rfpBC* mutants, transcript levels for carbohydrate transporters, glycolysis, the oxidative pentose phosphate pathway and the TCA cycle either decrease or do not change dramatically when cells are shifted from WL to FRL for 48 h (Supplementary Tables S3, S5). During the transition from WL to FRL, cells apparently experience short-term (WT) or perhaps even longer-term (*rfpABC* mutants) energy and reductant limitation. Collectively, the transcriptional profiling data indicate that at least some of the energy and reducing power, as well as some of the fixed carbon, nitrogen, and precursor metabolites required for remodeling of the photosynthetic apparatus, probably come from the degradation of proteins (e.g., WL-produced PBPs and possibly photosystem complexes) and the β-oxidation of fatty acids (reduction in total thylakoid membranes) but less from the breakdown of glycogen or polysaccharides. This may reflect the fact that photosynthesis is initially limited when cells are shifted from WL to FRL.

Thylakoid membranes are lipid bilayer membranes that are densely packed with protein complexes (Liu, 2016). Similar observations concerning reduction of the PBP content of cells in FRL are seen for *Leptolyngbya* sp. JSC-1 (Gan et al., 2014) and *H. hongdechloris* (Chen et al., 2012; Li et al., 2014). The lower total Chl content per cell indicates that there is less photosynthetic apparatus per cell, and in particular that there are probably fewer PSI complexes per cell, because most of the Chls are associated with PSI in cyanobacteria (Fujita and Murakami, 1987). The levels of PBPs and Chls are also lower in the *rfpB* and *rfpC* mutants of *Synechococcus* 7335, although slightly less so than in the WT (Ho et al., 2017a,c). Overall, the transcript abundance differences for proteases and enzymes for β-oxidation of fatty acids, as well as increased transcript levels for the *nblA* genes noted above, suggest that cells acclimating to FRL probably actively degrade some PBP and PSI complexes during the period when the photosynthetic apparatus is being remodeled.

**FIGURE 9:**
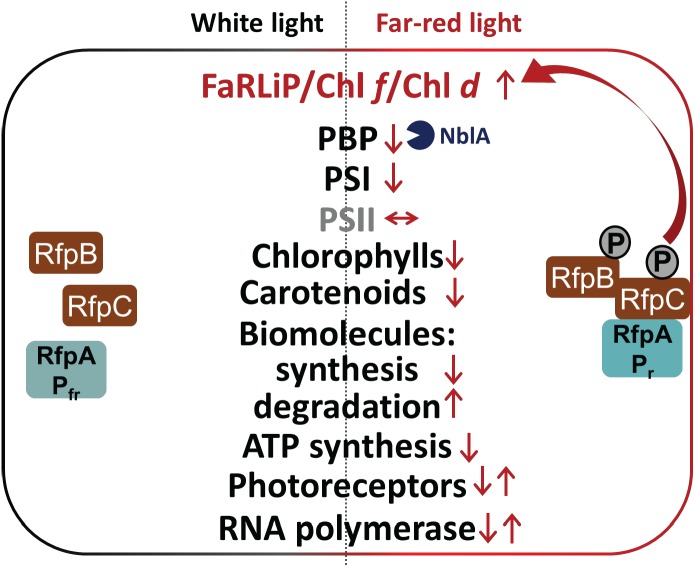
Summary of changes in relative transcription levels for selected gene categories in *C. fritschii* 9212 cells in WL and FRL. Small upward arrows indicate that relative transcript levels are generally higher and downward arrows indicate lower relative transcript abundances. Paired upward and downward arrows indicate that specific genes in these categories show increases or decreases, respectively. Increased transcript levels for *nblA* in FRL probably lead to higher NblA protein levels, which further contributes to lowering of PBP levels in cells grown in FRL by enhancing degradation of PBPs in FRL (blue Pacman). The RfpABC regulators activate transcription of the genes in the FaRLiP cluster, including the genes encoding these three proteins, in FRL as shown indicated by the two arrows. Most other responses to FRL are independent of RfpABC regulation. In FRL *C. fritschii* 9212 WT cells have transcriptional responses to reduce their overall rates of photosynthesis and growth, anabolism, and energy production, while enhancing the degradation of lipids and fatty acids and proteins to produce energy, reducing power and precursor metabolites.

Except for the genes in FaRLiP gene cluster, the transcriptional changes that occur when *C. fritschii* 9212 is shifted from WL to FRL appear to be largely independent of regulation by the RfpABC regulators. RfpABC-independent responses could be regulated at the transcriptional level by specific transcription factors, by changes in RNA polymerase sigma factor utilization, and possibly by other photoreceptors. In addition to RfpA, at least 31 other phytochromes and cyanobacteriochromes are encoded in the *C. fritschii* 9212 genome (Supplementary Tables S3, S5). Relative transcript levels for most of these potential photoregulatory proteins are unchanged in response to a shift from WL to FRL, and only *rfpA* shows strong differential transcription. Based on the transcriptional profiling analyses described above, some responses in *C. fritschii* 9212 upon transfer from WL to FRL for 48 h are summarized in Figure 9.

## Conclusion

In this study we definitively establish that the RfpABC regulatory proteins control Chl *d* synthesis as well as specific cellular responses involving the FaRLiP gene cluster (Figure 9). We document an extraordinary and initially confounding artifact by showing that addition of Em to WT or *rfp* mutants of *C. fritschii* 9212 leads to Chl *d* synthesis in cells grown in WL or FRL. Although the key catalyst/enzyme in Chl *d* biosynthesis is still unknown, our results strongly suggest that Chl *d* synthase is encoded within the FaRLiP gene cluster. We further demonstrate that thiol compounds or proteins with active-site cysteine residues could play a role in catalyzing Chl *d* formation. Finally, other physiological and metabolic responses to FRL seem to be regulated by photosensors other than RfpA, other sensor kinases, alternative sigma factors for RNA polymerase, other transcriptional regulators, or growth-rate-related transcriptional regulators. Because the FaRLiP response is weakly activated in the *rfpA* mutant when cells of this mutant are exposed to FRL for extended periods of time, it might be interesting in future studies to determine which red/far-red photoreceptor is responsible for this weaker, secondary activation of RfpC and RfpB.

## Data Availability

The datasets generated for this study can be found in NCBI GEO database, accession number GSE123329.

## Author Contributions

M-YH performed the research, analyzed the data, wrote the first draft of the manuscript, and participated in its revision. DB obtained the funding for the research, directed the research, assisted in interpretation of the results, and edited the manuscript. Both authors contributed to editing and correcting the final draft.

## Conflict of Interest Statement

The authors declare that the research was conducted in the absence of any commercial or financial relationships that could be construed as a potential conflict of interest.

## Footnotes

1 https://www.pasteur.fr/en/pcc

2 https://www.ushio-optosemi.com/jp/products/led/std/pdfs/lpm/L720-06AU.pdf

## Abbreviations

Chl: chlorophyll
Em: erythromycin
FaRLiP: far-red light photoacclimation
FRL: far-red light
HPLC: high-performance liquid chromatography
IAA: iodoacetamide
LED: light-emitting diode
PBS: phycobilisome
PSI: photosystem I
PSII: photosystem II
RP: reversed-phase
WL: white light
WT: wild type.

## References

[B1] Airs R. L. Temperton B. Sambles C. Farnham G. Skill S. C. Llewellyn C. A. (2014). Chlorophyll *f* and chlorophyll *d* are produced in the cyanobacterium *Chlorogloeopsis fritschii* when cultured under natural light and near-infrared radiation. *FEBS Lett.* 538 3770–3777. 10.1016/j.febslet.2014.08.026 25176411

[B2] Allakhverdiev S. I. Kreslavski V. D. Zhamukhamedov S. K. Voloshin R. A. Korol’kova D. V. Tomo T. (2016). Chlorophylls *d* and *f* and their role in primary photosynthetic process of cyanobacteria. *Biochemistry* 81 201–212. 10.1134/S0006297916030020 27262189

[B3] Averina S. Velichko N. Senatskaya E. Penevich A. (2018). Far-red light photoadaptations in aquatic cyanobacteria. *Hydrobiologica* 813 1–17. 10.1007/s10750-018-3519-x

[B4] Baier A. Winkler W. Korte T. Lockau W. Karradt A. (2014). Degradation of phycobilisomes in *Synechocystis* sp. PCC6803: evidence for essential formation of an NblA1/NblA2 heterodimer and its codegradation by a Clp protease complex. *J. Biol. Chem.* 289 11755–11766. 10.1074/jbc.M113.520601 24610785 PMC4002084

[B5] Behrendt L. Brejnrod A. Schliep M. Sørensen S. J. Larkum A. W. Kühl M. (2015). Chlorophyll *f*–driven photosynthesis in a cavernous cyanobacterium. *ISME J.* 9 2108–2011. 10.1038/ismej.2015.14 25668158 PMC4542031

[B6] Blankenship R. E. Chen M. (2013). Spectral expansion and antenna reduction can enhance photosynthesis for energy production. *Curr. Opin. Chem. Biol.* 17 457–461. 10.1016/j.cbpa.2013.03.031 23602382

[B7] Bryant D. A. (2016). *Prospects for Enhancing Plant and Algal Biofuel Production by Expanding the Wavelength Range for Photosynthesis.* Available at: https://vtechworks.lib.vt.edu/bitstream/handle/10919/78866/ISB-News-Report-Sep16.pdf?sequence=1.

[B8] Bryant D. A. Canniffe D. P. (2018). How Nature designs antenna proteins: design principles and functional realization of light-harvesting antenna systems in chlorophototrophic prokaryotes. *J. Phys. B Atom. Mol. Opt. Phys.* 51:033001 10.1088/1361-6455/aa9c3c

[B9] Chen M. (2014). Chlorophyll modifications and their spectral extension in oxygenic photosynthesis. *Annu. Rev. Biochem.* 83 317–340. 10.1146/annurev-biochem-072711-162943 24635479

[B10] Chen M. Blankenship R. E. (2011). Expanding the solar spectrum used by photosynthesis. *Trends Plant Sci.* 16 427–431. 10.1016/j.tplants.2011.03.011 21493120

[B11] Chen M. Li Y. Birch D. Willows R. D. (2012). A cyanobacterium that contains chlorophyll *f* – a red-absorbing photopigment. *FEBS Lett.* 586 3249–3254. 10.1016/j.febslet.2012.06.045 22796191

[B12] Chen M. Schliep M. Willows R. D. Cai Z.-L. Neilan B. A. Scheer H. (2010). A red-shifted chlorophyll. *Science* 329 1318–1319. 10.1126/science.1191127 20724585

[B13] Dubbs J. M. Bryant D. A. (1991). Molecular cloning and transcriptional analysis of the *cpeBA* operon of the cyanobacterium *Pseudanabaena* species PCC 7409. *Mol. Microbiol.* 5 3073–3085. 10.1111/j.1365-2958.1991.tb01867.x 1809846

[B14] Fujita Y. Murakami A. (1987). Regulation of electron transport composition in cyanobacterial photosynthetic system: stoichiometry among photosystem I and II complexes and their light-harvesting antennae and cytochrome *b*_6_/*f* complex. *Plant Cell Physiol.* 28 1547–1553. 10.1093/oxfordjournals.pcp.a077449

[B15] Fukusumi T. Matsuda K. Mizoguchi T. Miyatake T. Ito S. Ikeda T. (2012). Non-enzymatic conversion of chlorophyll-*a* into chlorophyll-*d* in vitro: a model oxidation pathway for chlorophyll-*d* biosynthesis. *FEBS Lett.* 586 2338–2341. 10.1016/j.febslet.2012.05.036 22659188

[B16] Gan F. Bryant D. A. (2015). Adaptive and acclimative responses of cyanobacteria to far-red light. *Environ. Microbiol.* 17 3450–3465. 10.1111/1462-2920.12992 26234306

[B17] Gan F. Shen G. Bryant D. A. (2015). Occurrence of far-red light photoacclimation (FaRLiP) in diverse cyanobacteria. *Life* 5 4–24. 10.3390/life5010004 25551681 PMC4390838

[B18] Gan F. Zhang S. Rockwell N. C. Martin S. S. Lagarias J. C. Bryant D. A. (2014). Extensive remodeling of a cyanobacterial photosynthetic apparatus in far-red light. *Science* 345 1312–1317. 10.1126/science.1256963 25214622

[B19] Gómez-Lojero C. Leyva-Carillo L. E. Herrera-Salgado P. Barrera-Rojas J. Ríos-Castro E. Gutiérrez-Cirlos E. B. (2018). *Leptolyngbya* CCM4, a cyanobacterium with far-red photoacclimation from Cuatro Ciénegas Basin, México. *Photosynthetica* 56 342–353. 10.1007/s11099-018-0774-z

[B20] Hirose Y. Fujisawa T. Ohtsubo Y. Katayama M. Misawa N. Wakazuki S. (2016). Complete genome sequence of cyanobacterium *Fischerella* sp. *NIES-*3754, providing thermoresistant optogenetic tools. *J. Biotechnol.* 220 45–46. 10.1016/j.jbiotec.2016.01.011 26784989

[B21] Ho M.-Y. (2018). *Characterization of Far-Red Light Photoacclimation in Cyanobacteria.* Doctoral dissertation, The Pennsylvania State University, University Park.

[B22] Ho M.-Y. Gan F. Shen G. Zhao C. Bryant D. A. (2017a). Far-red light photoacclimation (FaRLiP) in *Synechococcus* sp. PCC 7335: I. Regulation of FaRLiP gene expression. *Photosynth. Res.* 131 173–186. 10.1007/s11120-016-0309-z 27638320

[B23] Ho M.-Y. Gan F. Shen G. Bryant D. A. (2017b). Far-red light photoacclimation (FaRLiP) in *Synechococcus* sp. PCC 7335. II. Characterization of phycobiliproteins produced during acclimation to far-red light. *Photosynth. Res.* 131 187–202. 10.1007/s11120-016-0303-5 27623780

[B24] Ho M.-Y. Soulier N. T. Canniffe D. P. Shen G. Bryant D. A. (2017c). Light regulation of pigment and photosystem biosynthesis in cyanobacteria. *Curr. Opin. Plant. Biol.* 37 24–33. 10.1016/j.pbi.2017.03.006 28391049

[B25] Ho M.-Y. Shen G. Canniffe D. P. Zhao C. Bryant D. A. (2016). Light-dependent chlorophyll *f* synthase is a highly divergent paralog of PsbA of Photosystem II. *Science* 353:aaf9178. 10.1126/science.aaf9178 27386923

[B26] Karradt A. Sobanski J. Mattow J. Lockau W. Baier K. (2008). NblA, a key protein of phycobilisome degradation, interacts with ClpC, a HSP100 chaperone partner of a cyanobacterial Clp protease. *J. Biol. Chem.* 283 32394–32403. 10.1074/jbc.M805823200 18818204

[B27] Kerfeld C. A. Sawaya M. R. Brahmandam V. Cascio D. Ho K. K. Trevithick-Sutton C. C. (2003). The crystal structure of a cyanobacterial water-soluble carotenoid binding protein. *Structure* 11 55–65. 10.1016/S0969-2126(02)00936-X 12517340

[B28] Kirolovsky D. Kerfeld C. A. (2013). The orange carotenoid protein: a blue-green light photoactive protein. *Photochem. Photobiol. Sci.* 12 1135–1143. 10.1039/c3pp25406b 23396391

[B29] Kirolovsky D. Kerfeld C. A. (2016). Cyanobacterial photoprotection by the orange carotenoid protein. *Nat. Plants* 2:16180. 10.1038/nplants.2016.180 27909300

[B30] Koizumi H. Itoh Y. Hosoda S. Akiyama M. Hoshino T. Shiraiwa Y. (2005). Serendipitous discovery of Chl *d* formation from Chl *a* with papain. *Sci. Tech. Adv. Mater.* 6 551–557. 10.1016/j.stam.2005.06.022

[B31] Kurashov V. Ho M.-Y. Shen G. Piedl K. Laremore T. N. Bryant D. A. (2019). Energy transfer from chlorophyll *f* to the trapping center in naturally occurring and engineered photosystem I complexes. *Photosynth. Res.* 10.1007/s11120-019-00616-x [Epub ahead of print]. 30710189

[B32] Li H. Durbin R. (2009). Fast and accurate short read alignment with Burrows–Wheeler transform. *Bioinformatics* 25 1754–1760. 10.1093/bioinformatics/btp324 19451168 PMC2705234

[B33] Li Y. Lin Y. Garvey C. J. Birch D. Corkery R. W. Loughlin P. C. (2016). Characterization of red-shifted phycobilisomes isolated from the chlorophyll *f*-containing cyanobacterium *Halomicronema hongdechloris*. *Biochim. Biophys. Acta* 1857 107–114. 10.1016/j.bbabio.2015.10.009 26514405

[B34] Li Y. Lin Y. Loughlin P. C. Chen M. (2014). Optimization and effects of different culture conditions on growth of *Halomicronema hongdechloris* – a filamentous cyanobacterium containing chlorophyll *f*. *Front. Plant Sci.* 5:67. 10.3389/fpls.2014.00067 24616731 PMC3934312

[B35] Li Y. Vella N. Chen M. (2018). Characterization of isolated photosystem I from *Halomicronema hongdechloris*, a chlorophyll *f*-producing cyanobacterium. *Photosynthetica* 56 306–315.

[B36] Liu L.-N. (2016). Distribution and dynamics of electron transport complexes in cyanobacterial thylakoid membranes. *Biochim. Biophys. Acta* 1857 256–265. 10.1016/j.bbabio.2015.11.010 26619924 PMC4756276

[B37] López-Igual R. Wilson A. Leverenz R. L. Melnicki M. R. Bourcier De Carbon C. Sutter M. (2016). Different functions of the paralogs to the N-terminal domain of the orange carotenoid protein in the cyanobacterium *Anabaena* sp. PCC 7120. *Plant Physiol.* 171 1852–1866. 10.1104/pp.16.00502 27208286 PMC4936580

[B38] Loughlin P. C. Willows R. D. Chen M. (2014). In vitro conversion of vinyl to formyl groups in naturally occurring chlorophylls. *Sci. Rep.* 4:6069. 10.1038/srep06069 25119484 PMC4132379

[B39] Ludwig M. Bryant D. A. (2011). Transcription profiling of the cyanobacterium *Synechococcus* sp. PCC 7002 using high-throughput cDNA sequencing. *Front. Microbiol.* 2:41 10.3389/fmicb.2011.00041 PMC3133671 21779275

[B40] Ludwig M. Pandelia M. E. Cew C.-Y. Zhang B. Golbeck J. H. Krebs C. (2014). ChlR protein of *Synechococcus* sp. PCC 7002 is a transcription activator that uses an oxygen-sensitive [4Fe-4S] cluster to control genes involved in pigment biosynthesis. *J. Biol. Chem.* 289 16624–16639. 10.1074/jbc.M114.561233 24782315 PMC4059106

[B41] Magnuson A. Krassen H. Stensjö K. Ho F. M. Styring S. (2011). Modeling photosystem I with the alternative reaction center protein PsaB2 in the nitrogen fixing cyanobacterium *Nostoc punctiforme*. *Biochim. Biophys. Acta* 1807 1152–1161. 10.1016/j.bbabio.2011.05.004 21605545

[B42] Melnicki M. R. Leverenz R. L. Sutter M. López-Igual R. Wilson A. Pawlowski E. G. (2016). Structure, diversity, and evolution of a new family of soluble carotenoid-binding proteins in cyanobacteria. *Mol. Plant* 9 1379–1394. 10.1016/j.molp.2016.06.009 27392608

[B43] Miyashita H. Ikemoto H. Kurano N. Adachi K. Chihara M. Miyachi S. (1996). Chlorophyll *d* as a major pigment. *Nature* 383:402. 10.1038/383402a0 14747075

[B44] Miyashita H. Ohkubo S. Komatus H. Sorimachi Y. Fukayama D. Fujinama D. (2014). Discovery of chlorophyll *d* in *Acaryochloris marina* and chlorophyll *f* in a unicellular cyanobacterium, strain KC1, isolated from Lake Biwa. *J. Phys. Chem. Biophys.* 4:149 10.4172/2161-0398.1000149

[B45] Mullineaux C. W. (2014). Co-existence of photosynthetic and respiratory activities in cyanobacterial thylakoid membranes. *Biochim. Biophys. Acta* 1837 503–511. 10.1016/j.bbabio.2013.11.017 24316145

[B46] Nowack S. Olsen M. T. Schaible G. A. Becraft E. D. Shen G. Klapper I. (2015). The molecular dimension of microbial species: 2. *Synechococcus* strains representative of putative ecotypes inhabiting different depths in the Mushroom Spring microbial mat exhibit different adaptive and acclimative responses to light. *Front. Microbiol.* 6:626. 10.3389/fmicb.2015.00626 26175719 PMC4484337

[B47] Nürnberg D. J. Morton J. Santabarbara S. Telfer A. Joliot P. Antonaru L. A. (2018). Photochemistry beyond the red limit in chlorophyll *f*–containing photosystems. *Science* 360 1210–1213. 10.1126/science.aar8313 29903971

[B48] Ohkubo S. Miyashita H. (2017). A niche for cyanobacteria producing chlorophyll *f* within a microbial mat. *ISME J.* 11 2368–2378. 10.1038/ismej.2017.98 28622287 PMC5607378

[B49] Olsen M. Nowack S. Wood J. Becraft E. Labutti K. Lipzen A. (2015). The molecular dimension of microbial species: 3. Comparative genomics of *Synechococcus* strains with different light responses and in situ diel transcription patterns of associated putative ecotypes in the Mushroom Spring microbial mat. *Front. Microbiol.* 6:604. 10.3389/fmicb.2015.00604 26157428 PMC4477158

[B50] Pfannschmidt T. Nilsson A. Allen J. F. (1999). Photosynthetic control of chloroplast gene expression. *Nature* 397:625.

[B51] Rippka R. Deruelles J. Waterbury J. B. Herdman M. Stanier R. Y. (1979). Generic assignments, strain histories and properties of pure cultures of cyanobacteria. *Microbiology* 111 1–61. 10.1099/00221287-111-1-1

[B52] Rockwell N. C. Su Y.-S. Lagarias J. C. (2006). Phytochrome structure and signaling mechanisms. *Annu. Rev. Plant Biol.* 57 837–858. 10.1146/annurev.arplant.56.032604.144208 16669784 PMC2664748

[B53] Schliep M. Crossett B. Willows R. D. Chen M. (2010). 18O labeling of chlorophyll *d* in *Acaryochloris marina* reveals that chlorophyll *a* and molecular oxygen are precursors. *J. Biol. Chem.* 285 28450–28456. 10.1074/jbc.M110.146753 20610399 PMC2937870

[B54] Shen G. Canniffe D. P. Ho M.-Y. Kurashov V. van der Est A. Golbeck J. H. (2019). Characterization of chlorophyll *f* synthase heterologously produced in *Synechococcus* sp. PCC 7002. *Photosyn. Res.* 10.1007/s11120-018-00610-9 30607859

[B55] Smythe C. V. (1936). The reaction of iodoacetate and of iodoacetamide with various sulfhydryl groups, with urease, and with yeast preparations. *J. Biol. Chem.* 114 601–612.

[B56] Sutter M. Wilson A. Leverenz R. L. Lopez-Igual R. Thurotte A. Salmeen A. E. (2013). Crystral structure of the FRP and identification of the active site for modulation of OCP-mediated photoprotection in cyanobacteria. *Proc. Natl. Acad. Sci. U.S.A.* 110 10022–10027. 10.1073/pnas.1303673110 23716688 PMC3683793

[B57] Trampe E. Kühl M. (2016). Chlorophyll *f* distribution and dynamics in cyanobacterial beachrock biofilms. *J. Phycol.* 52 990–996. 10.1111/jpy.12450 27439961

[B58] Yoneda A. Wittmann B. J. King J. D. Blankenship R. E. Dantas G. (2016). Transcriptomic analysis illuminates genes involved in chlorophyll synthesis after nitrogen starvation in *Acaryochloris* sp. *CCMEE* 5410. *Photosynth. Res.* 129 171–182. 10.1007/s11120-016-0279-1 27276888

[B59] Zhao C. Gan F. Shen G. Bryant D. A. (2015). RfpA, RfpB, and RfpC are the master control elements of Far-Red Light Photoacclimation (FaRLiP). *Front. Microbiol.* 6:1303. 10.3389/fmicb.2015.01303 26635768 PMC4658448

