# Supplementary material for: Global Transcriptional Profiling of the Cyanobacterium Chlorogloeopsis fritschii PCC 9212 in Far-Red Light: Insights Into the Regulation of Chlorophyll d Synthesis
Source: Front Microbiol. 2019 Mar 13;10:465. doi: 10.3389/fmicb.2019.00465 (PMC6424891; doi:10.3389/fmicb.2019.00465)
Supplement: Supplementary file 1 [file Table_1.DOCX]

**Table S1.** Summary statistics for sequences obtained from RNA sequencing analysis of the indicated samples from cells grown in a medium containing erythromycin or in the absence of erythromycin in WL and FRL

| **Sample**^1^ | **Total reads** | **Mapped reads (excluding rRNA)** | **Percent rRNA (%)** | **Uniquely mapped reads**  **(excluding rRNA)** | **Unique reads (%)** |
| --- | --- | --- | --- | --- | --- |
| **Experiment One** | | | | | |
| **WT WL** | 7,494,625 | 7,273,410 | 3.0% | 7,215,800 | 99.2% |
| ***rfpA* WL +Em** | 4,342,674 | 4,268,374 | 1.7% | 4,197,983 | 98.4% |
| ***rfpB* WL +Em** | 6,888,313 | 4,272,322 | 38.0% | 4,217,866 | 98.7% |
| ***rfpC* WL +Em** | 8,008,522 | 5,516,130 | 31.1% | 5,469,680 | 99.2% |
| **WT FRL** | 7,402,914 | 7,098,795 | 4.1% | 7,058,657 | 99.4% |
| ***rfpA* FRL +Em** | 7,312,573 | 7,200,422 | 1.5% | 7,173,890 | 99.6% |
| ***rfpB* FRL +Em** | 6,272,006 | 3,479,124 | 44.5% | 3,468,947 | 99.7% |
| ***rfpC* FRL +Em** | 6,489,711 | 4,894,832 | 24.6% | 4,832,598 | 98.7% |
| **Experiment Two** | | | | | |
| **WT WL** | 47,695,298 | 21,288,101 | 55.4% | 21,232,390 | 99.7% |
| **WT WL +Em^2^** | 22,857,425 | 10,442,045 | 54.3% | 10,283,518 | 98.5% |
| ***rfpB* WL** | 47,695,298 | 21,659,076 | 54.6% | 21,599,816 | 99.7% |
| ***rfpC* WL** | 25,440,939 | 12,055,979 | 52.6% | 12,022,941 | 99.7% |
| **WT FRL** | 47,072,319 | 19,298,837 | 59.0% | 19,234,148 | 99.7% |
| **WT FRL +Em^2^** | 56,112,279 | 26,614,820 | 52.6% | 26,449,937 | 99.4% |
| ***rfpB* FRL** | 51,214,300 | 21,415,732 | 58.2% | 21,341,872 | 99.7% |
| ***rfpC* FRL** | 57,350,699 | 24,355,052 | 57.5% | 24,275,994 | 99.7% |

^1^Abbreviations: WL, white light; FRL, far-red light; Em, erythromycin.

^2^WT *C. fritschii* 9212 was grown in the presence of a sublethal concentration of erythromycin (5 µg ml^–1^) in the indicated light condition as described in the Materials and Methods.
