# Supplementary material for: Global Transcriptional Profiling of the Cyanobacterium Chlorogloeopsis fritschii PCC 9212 in Far-Red Light: Insights Into the Regulation of Chlorophyll d Synthesis
Source: Front Microbiol. 2019 Mar 13;10:465. doi: 10.3389/fmicb.2019.00465 (PMC6424891; doi:10.3389/fmicb.2019.00465)
Supplement: Supplementary file 6 [file Data_Sheet_1.PDF]

Supplementary Material

Global transcriptional profiling of the cyanobacterium *Chlorogloeopsis fritschii* PCC 9212 in far-red light: insights into the regulation of chlorophyll *d* synthesis

Ming-Yang Ho[1,2,†] and Donald A. Bryant[1,2,3]*

[1]Department of Biochemistry and Molecular Biology, and [2]Intercollege Graduate Degree Program in Plant Biology, The Pennsylvania State University, University Park, PA 16802, USA

[3]Department of Chemistry and Biochemistry, Montana State University, Bozeman, MT 59717 USA.

[†]**Current Address:** Plant Research Laboratory, Michigan State University, 612 Wilson Road, East Lansing, MI 48824-1319

***Correspondence:** Dr. Donald A. Bryant, S-002 Frear Laboratory, Department of Biochemistry and Molecular Biology, The Pennsylvania State University, University Park, PA 16802 USA. Phone; 814-865-1992; Fax: 814-863-7024; e-mail: dab14@psu.edu

Supplementary Tables S1 to S5

Table S1. Summary statistics for sequences obtained from RNA sequencing analysis of the indicated samples from cells grown in a medium containing erythromycin or in the absence of erythromycin in WL and FRL.

Table S2 Normalized transcript level of all genes in *C. fritschii* 9212 (WT without addition of erythromycin and *rfpA*, *rfpB*, and *rfpC* mutants supplemented with erythromycin) grown in WL or in FRL for 48 h.

Table S3 Normalized transcript level of all genes in *C. fritschii* 9212 (WT with and without addition of erythromycin and *rfpB* and *rfpC* mutants without addition of erythromycin) grown in WL or in FRL for 48 h. See Fig. S1 for the details concerning experimental design.

Table S4 Comparison of normalized transcript levels of genes related to photosynthesis in *C. fritschii* PCC 9212 (WT, and *rfpB* and *rfpC* mutants) grown in WL or FRL for 48 h. N/A stands for not available. This occurs when the denominator (the read count for a particular gene in WL) is zero. *p*-values were deleted for clarity.

Table S5 Comparison of normalized transcript levels of genes involved in metabolisms other than photosynthesis in *C. fritschii* PCC 9212 (WT, and *rfpB* and *rfpC* mutants) grown in WL or FRL for 48 h. N/A stands for not available. This occurs when the denominator (the read count for a particular gene in WL) is zero. *p*-values were deleted for clarity. Genes belonging to more than one category are indicated by *, #, and $, respectively.

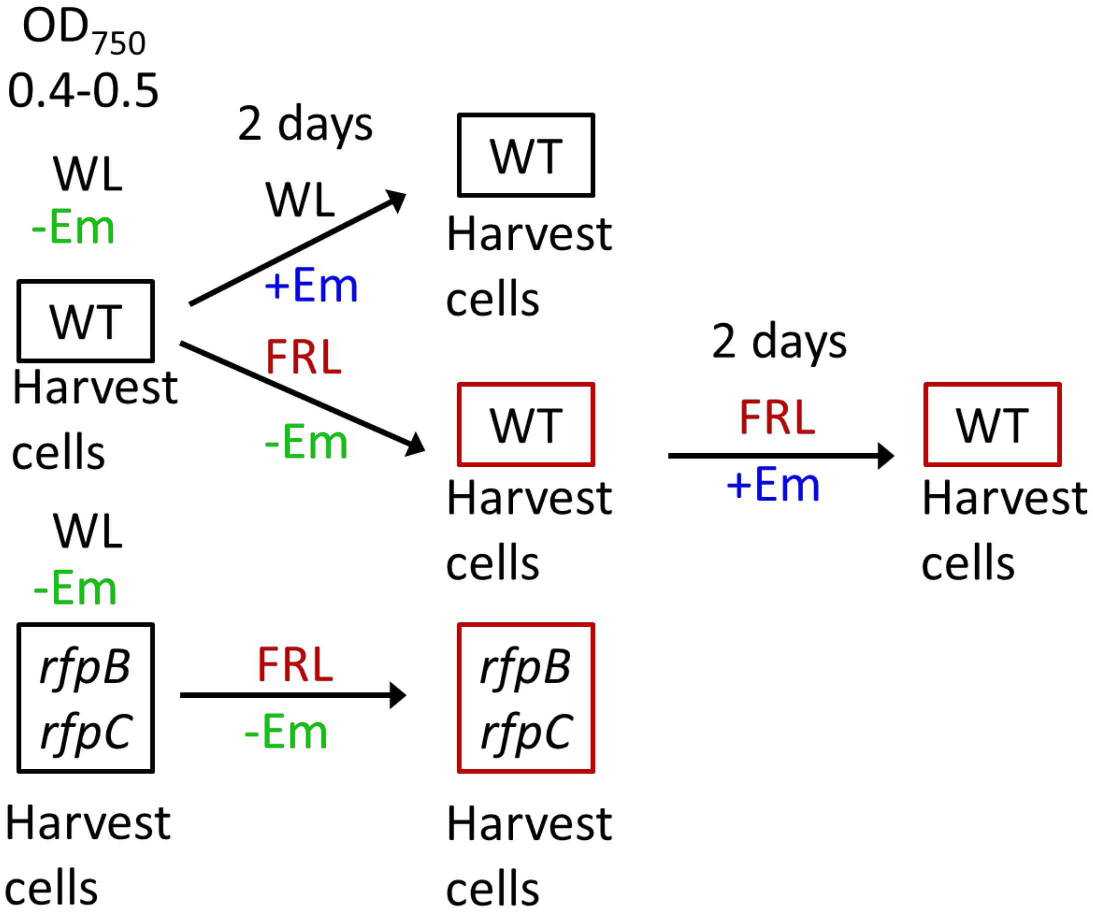

Figure S1. Scheme showing the experimental design of the second transcription profiling experiment. *C. fritschii* 9212 wild type (WT) and *rfpB* and *rfpC* mutants (*rfpB* and *rfpC*) were grown in WL without erythromycin (–Em) until the optical density at 750 (OD_{750}) reached 0.4-0.5. Cells were harvested and treated as indicated for two days. (WL: white light; FRL; far-red light; +Em: addition of 5 µg ml^{-1} of erythromycin in the medium). After two days, some cells were harvested, and erythromycin (5 µg ml^{-1}) was added to WT cells cultured in FRL. The WT cells were grown in FRL for two more days before the cells were harvested.

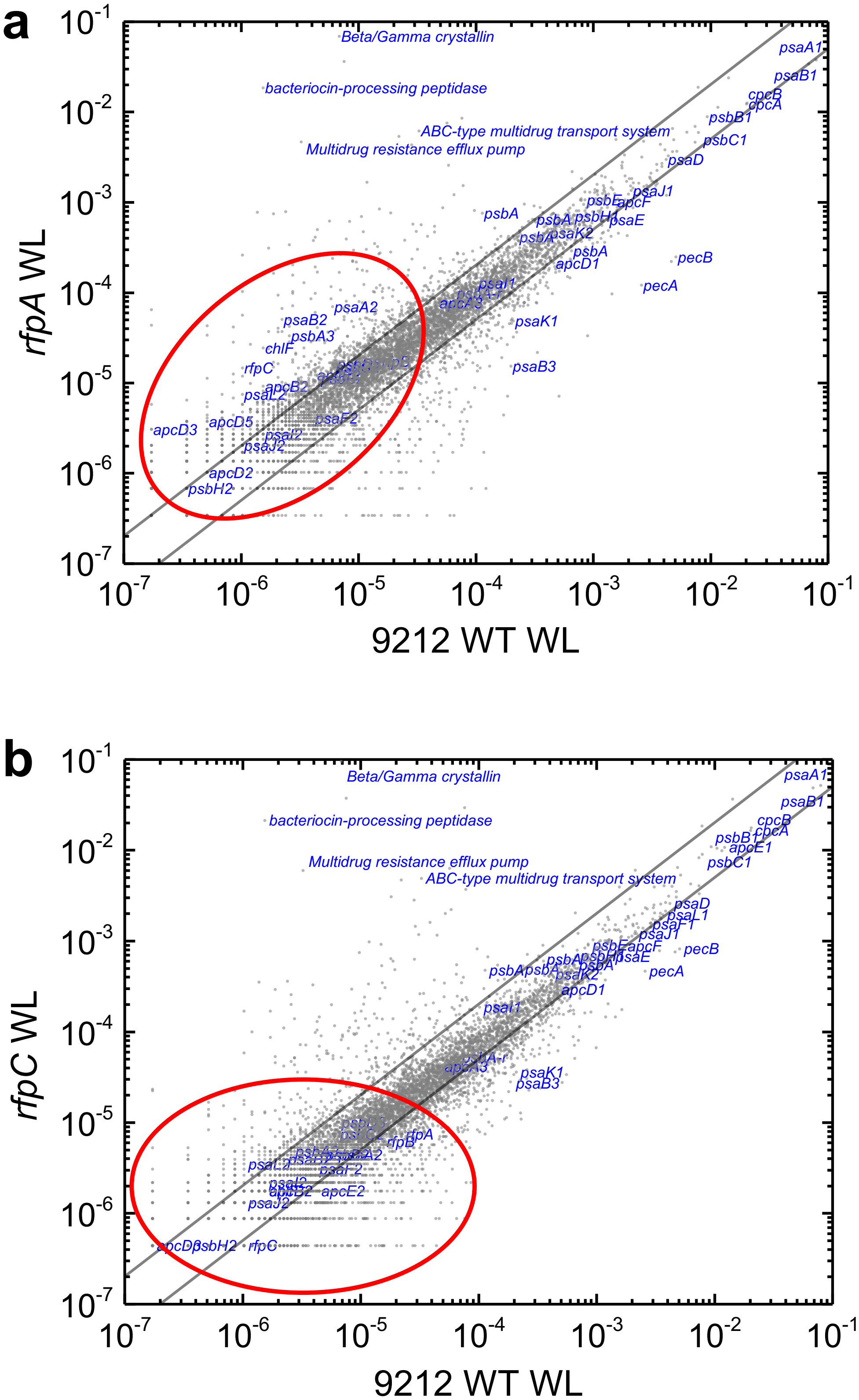
a
rfpA WL
9212 WT WL
Beta/Gamma crystallin
bacteriocin-processing peptidase
ABC-type multidrug transport system
Multidrug resistance efflux pump
psaA1
psaB1
cpcB
cpcA
psbB1
psbC1
psaD
psaJ1
apcF
psaE
psbA
psaK2
apcD1
pecB
pecA
psaI1
apcA3
psaK1
psaB3
psaA2
psaB2
psbA3
chlF
rfpC
apcB2
psaL2
psaF2
apcD3
apcD5
psaI2
psaJ2
apcD2
psbH2
b
rfpC WL
9212 WT WL
Beta/Gamma crystallin
bacteriocin-processing peptidase
Multidrug resistance efflux pump
ABC-type multidrug transport system
psaA1
psaB1
cpcB
cpcA
psbB1
apcE1
psbC1
psaD
psaL1
psaF1
psaJ1
pecB
apcF
psaE
pecA
psaK2
apcD1
psaI1
apcA3
psaK1
psaB3
rfpA
rfpB
psaF2
psaI2
apcB2
apcE2
psaJ2
psbH2
rfpC

Figure S2. Scatter plot showing a comparison of relative transcript abundances in cells of the *rfpA* and *rfpC* mutants and WT strains of *C. fritschii* 9212 grown in WL. The X- and Y-axes represent normalized transcript abundances in **a** the *rfpA* mutant and the WT grown in WL and **b** the *rfpC* mutant and the WT grown in WL, respectively. Each gray dot represents one gene. Genes of interest, including genes in FaRLiP gene cluster (red oval) and genes encoding PSI, PSII, and PBP/PBS subunits are labeled in blue. The paired gray diagonals indicate the thresholds for a two-fold increase and a 50% reduction in relative transcript abundance, respectively.

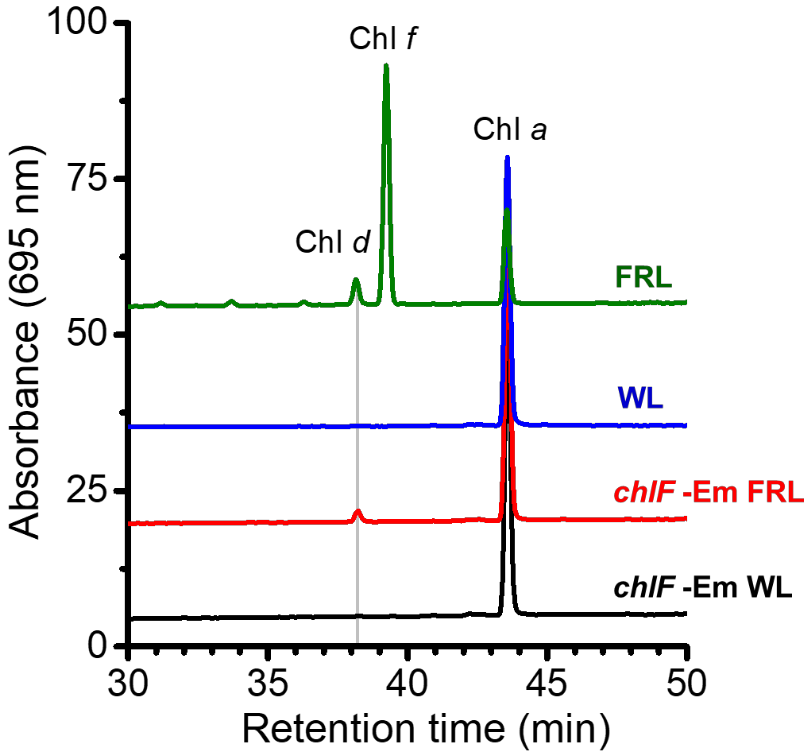

Figure S3. RP-HPLC elution profiles of pigments extracted from the *chlF* mutant in *C. fritschii* 9212 showing Chl *d* synthesis is independent to Chl *f* synthesis. Pigment extract from *C. fritschii* 9212 WT cells grown in WL (WL) or from cells acclimated for more than one month in FRL (FRL) were used as standards for Chl *a*, Chl *d*, and Chl *f*. Cells of the *chlF* mutant of *C. fritschii* 9212 were cultured without erythromycin in WL (*chlF* –Em WL) and FRL (*chlF* –Em FRL), and pigments were extracted and separated through reversed-phase HPLC. Please see Ho et al. (2016) for a more detailed characterization of the *chlF* mutant.

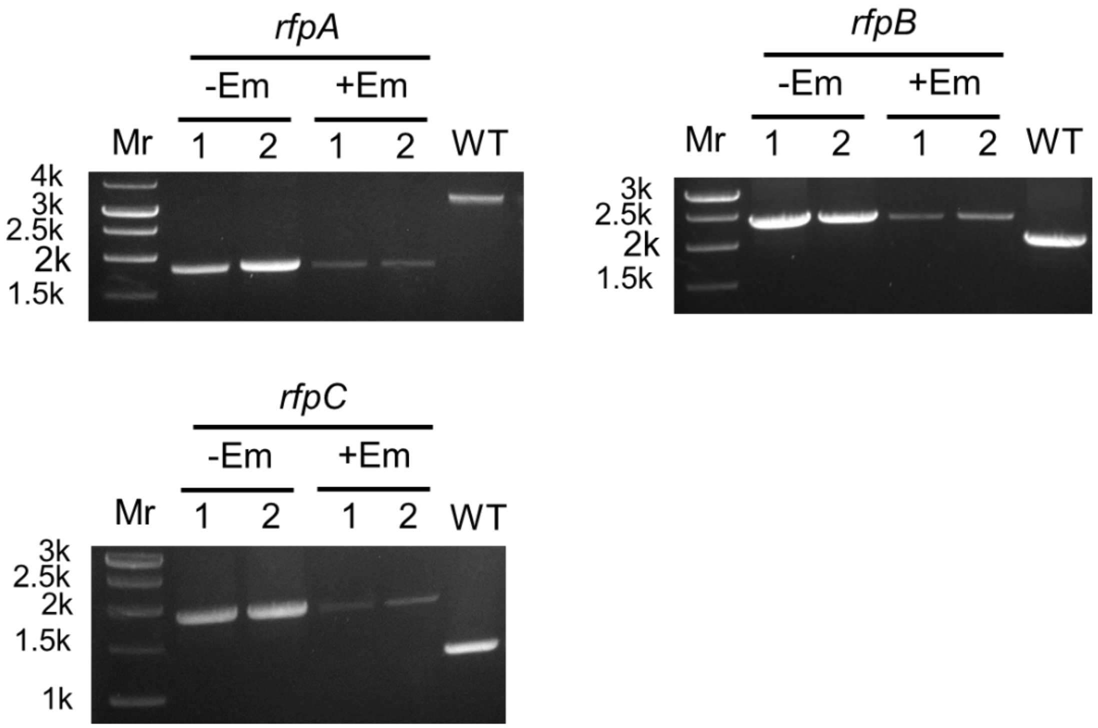

Figure S4. Polymerase chain reaction (PCR) results to validate the absence of WT cells of *C. fritschii* 9212 in cultures grown without erythromycin. PCR analyses were performed for aliquots of cells derived from cultures used for pigmentation analysis of the *rfpA*, *rfpB*, and *rfpC* mutants (see **Fig. 5b**) by using the gene-specific PCR primers described in Zhao et al. (2015). Mutants cultured with or without erythromycin are indicated as (+Em) and (–Em). *C. fritschii* PCC 9212 WT cells were used as negative controls for PCR. Mr represents DNA ladder, and the sizes of fragments are labeled on the left of each image. For each mutant, the amplicons for the *rfpA*, *rfpB*, and *rfpC* genes confirm that the –Em or +Em cultures were not contaminated with WT cells. Lanes 1 and 2 for each condition show amplicons derived from two independent cultures.

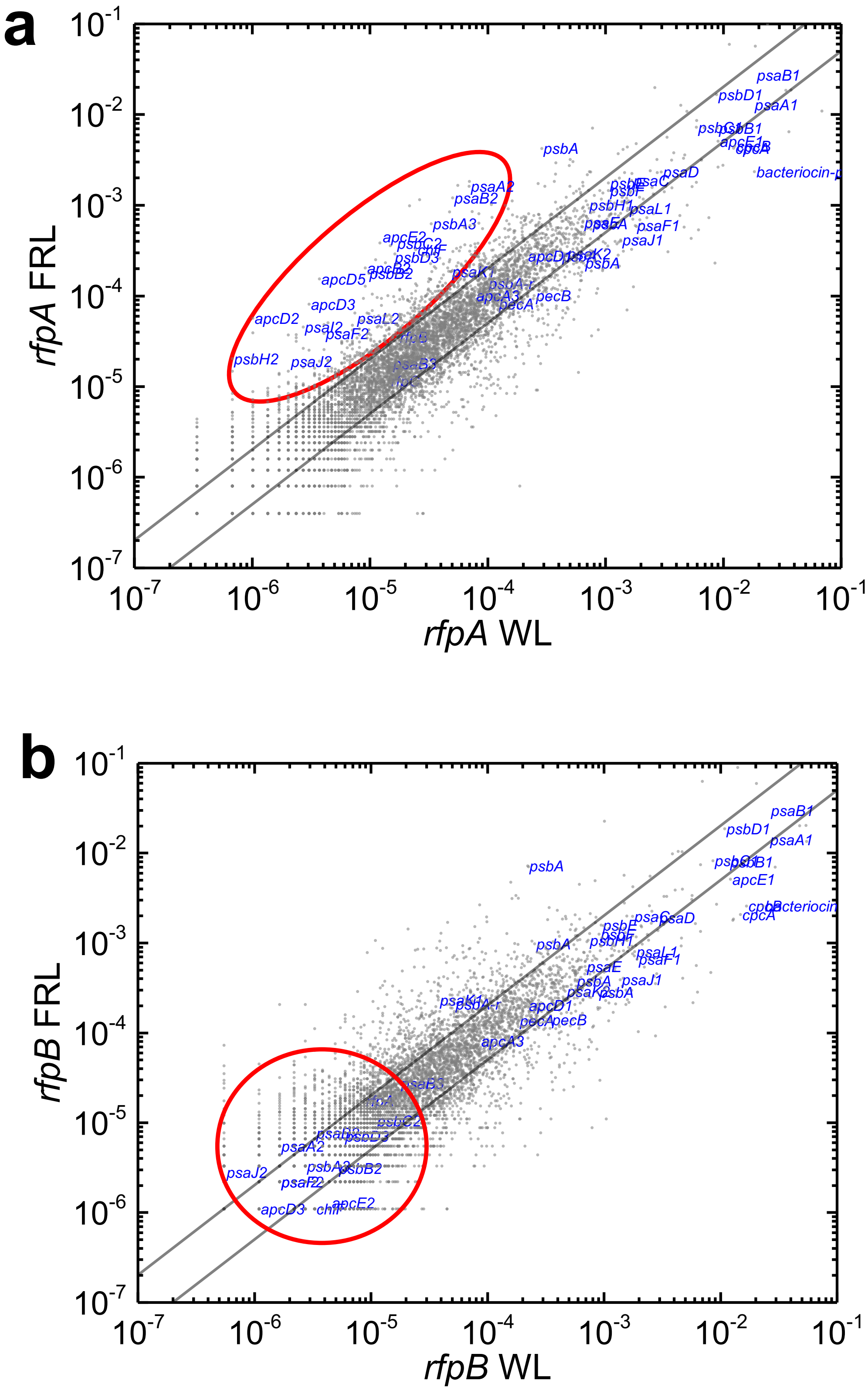
a
rfpA FRL
rfpA WL
10-1
10-2
10-3
10-4
10-5
10-6
10-7
psaB1
psbD1
psaA1
psbB1
bacteriocin-
psbA
psaD
psaA2
psaB2
psbA3
psbH1
psaL1
psaF1
psaJ1
apcE2
psbD3
apcD5
psbB2
psaK1
psaK2
apcD1
apcD3
apcD2
psaL2
psaF2
psbH2
psaJ2
apcA3
pecA
pecB
b
rfpB FRL
rfpB WL
psaB1
psbD1
psaA1
psbB1
apcE1
cpcA
psbA
psaD
psbH1
psaL1
psaF1
psaE
psaJ1
psaK1
apcD1
pecA
pecB
apcA3
psaB3
psaA2
psbD3
psbA2
psbB2
psaJ2
psaF2
apcD3
chlF
apcE2

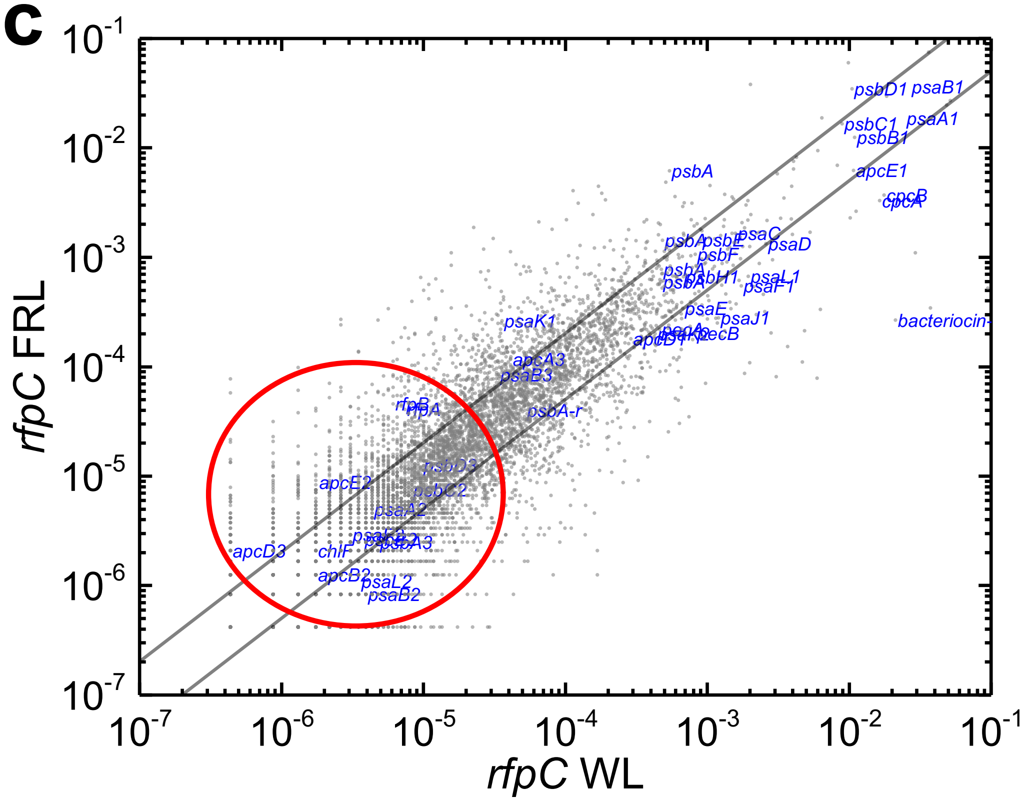

Figure S5. Scatter plots comparing relative transcript abundances in cells of the *rfpA*, *rfpB*, *and rfpC* mutant of *C. fritschii* PCC 9212 grown in FRL and WL. The X- and Y-axes represent normalized transcript abundances in the **a** *rfpA* mutant, **b** *rfpB* mutant, and **c** *rfpC* mutant grown in FRL and in WL, respectively. Each gray dot represents one gene. Genes of interest, including genes in FaRLiP gene cluster (red ovals) and genes encoding PSI, PSII, and PBP/PBS subunits are labeled in blue. The paired gray diagonals indicate the thresholds for a two-fold increase and a 50% reduction in relative transcript abundance, respectively.

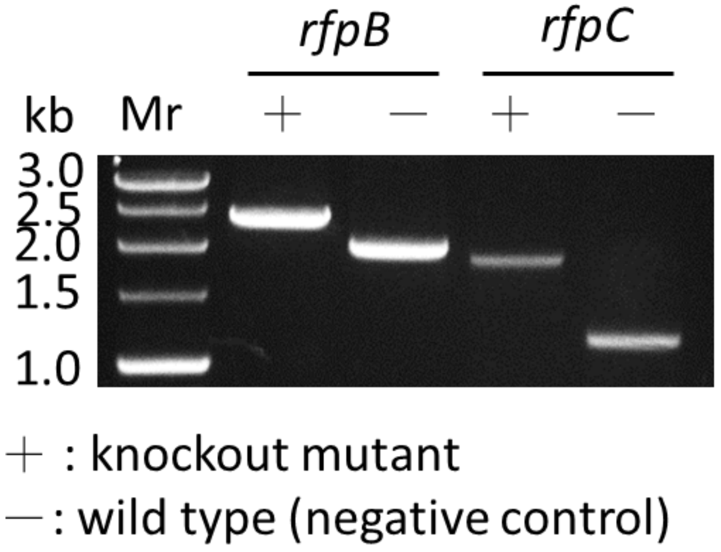

Figure S6. PCR results to validate the absence of WT cells of *C. fritschii* 9212 in cultures grown without erythromycin. PCR analyses were performed upon aliquots of cells derived from cultures used for transcriptomic analysis of the *rfpB* and *rfpC* mutants (see **Figs. 7, 8, and S8**) by using the gene-specific PCR primers described in Zhao et al. (2015). *C. fritschii* 9212 WT cells were used as negative controls for PCR. Mr represents DNA ladder, and the sizes of fragments are labeled on the left of the image. For each mutant, the amplicons for the *rfpB*, and *rfpC* genes confirm that the cultures were not contaminated with WT cells.

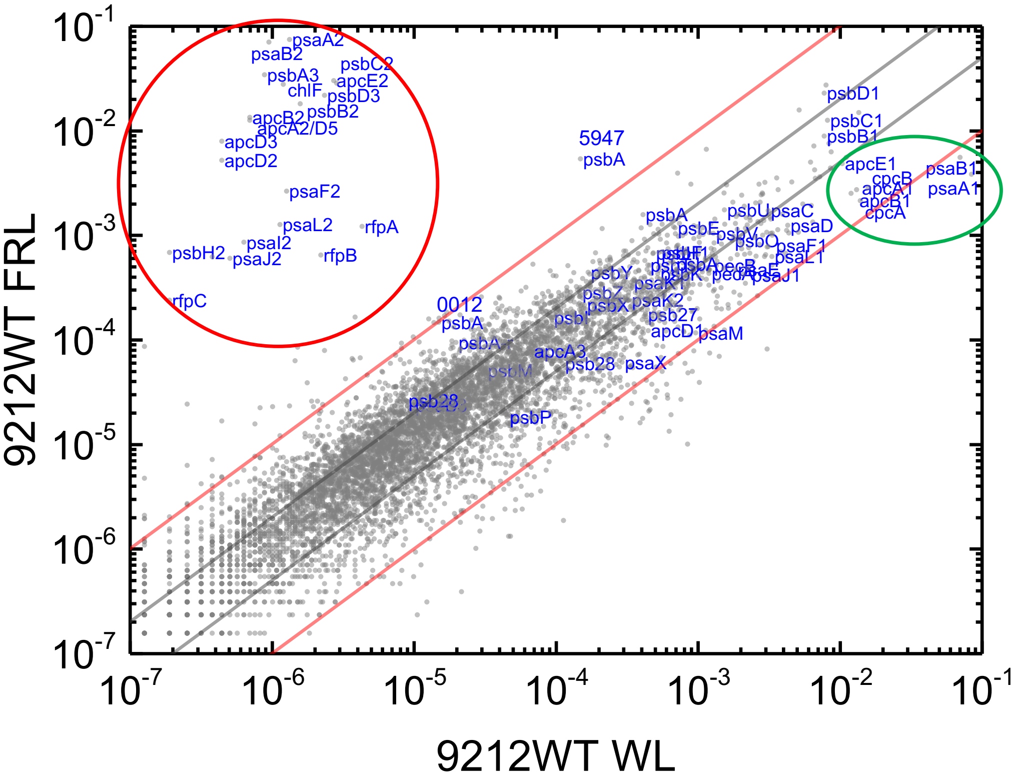

Figure S7. Scatter plot showing a comparison of relative transcript abundances for *C. fritschii* PCC 9212 cells grown in FRL and WL. This is a second set of transcriptomic results performed with *rfpB* and *rfpC* without erythromycin. The conditions of *C. fritschii* 9212 WT cells are identical as described in **Fig. 2**. Two *psbA* genes (UYEDRAFT_00012 and UYEDRAFT_05947) are labeled as 0012 and 5947, respectively. The paired gray and red diagonals indicate the thresholds for a two-fold and ten-fold increase and a 50% and 10% reduction in relative transcript abundance, respectively. The red oval indicates the genes of the FaRLiP gene cluster, and the green oval indicates selected PSI and PBS genes whose products occur in the photosynthetic apparatus produced in WL.

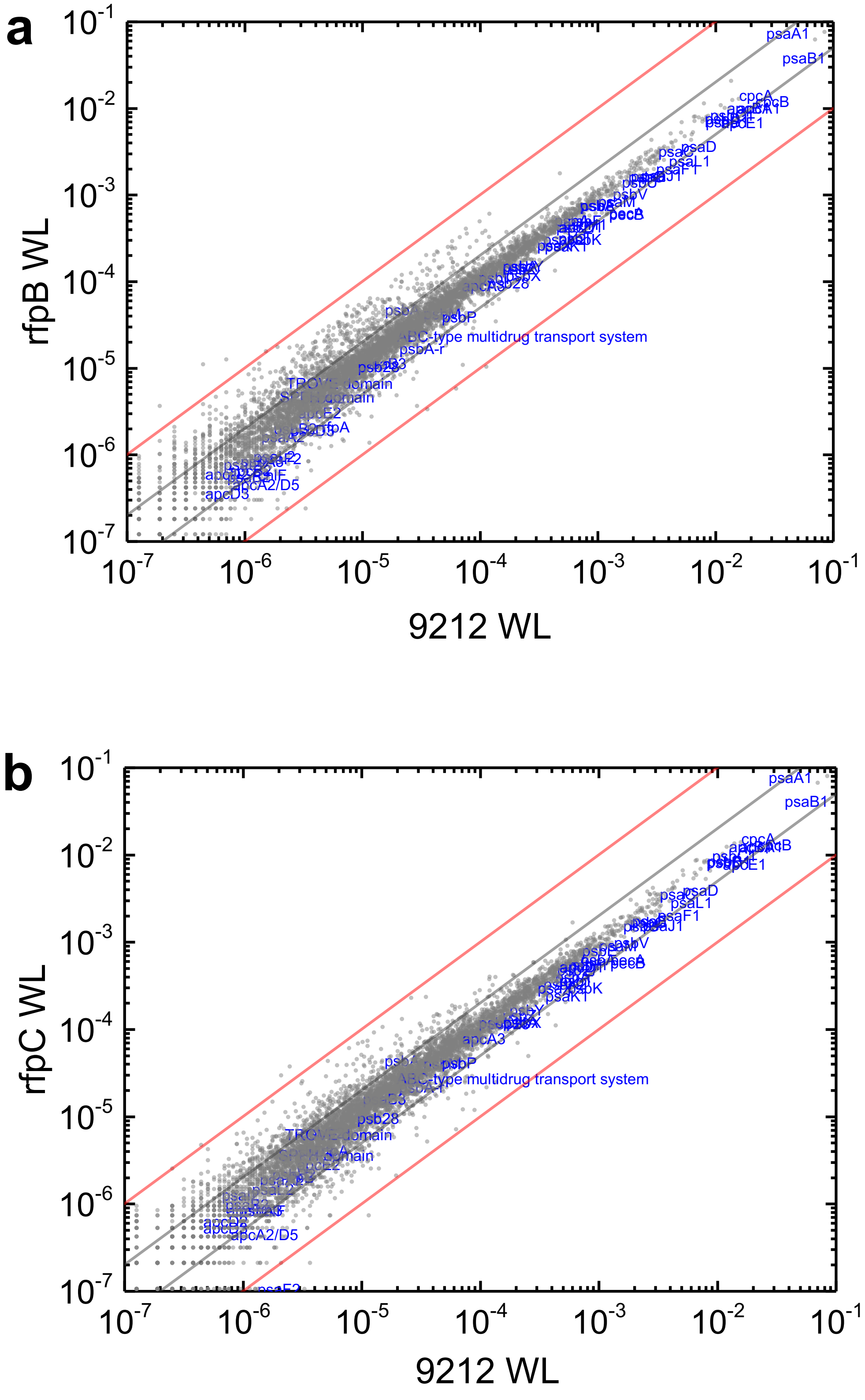
a
rfpB WL
9212 WL
psaA1
psaB1
cpcA
psaD
psaL1
psbV
pecB
psbP
ABC-type multidrug transport system
psbA-r
psb28
apcE2
apcA2/D5
apcD3
b
rfpC WL
9212 WL
psaA1
psaB1
cpcA
psaD
psaL1
psbV
pecB
psaK1
apcA3
psbP
ABC-type multidrug transport system
psb28
apcE2
apcA2/D5

Figure S8. Scatter plots comparing relative transcript abundances in cells of the WT and *rfpB* and *rfpC* mutants of *C. fritschii* 9212 grown in WL. The X- and Y-axes represent normalized transcript abundances in the WT and **a** *rfpB* mutant or **b** *rfpC* mutant grown in WL, respectively. Each gray dot represents one gene. Genes of interest, including genes in FaRLiP gene cluster and genes encoding PSI, PSII, and PBP/PBS subunits are labeled in blue. The paired gray and red diagonals indicate the thresholds for a two-fold and ten-fold increase and a 50% and 10% reduction in relative transcript abundance, respectively.
